# Neural mechanisms of costly helping in the general population and mirror-pain synesthetes

**DOI:** 10.1038/s41598-024-62422-3

**Published:** 2024-05-21

**Authors:** Kalliopi Ioumpa, Selene Gallo, Christian Keysers, Valeria Gazzola

**Affiliations:** 1 Netherlands Institute for Neuroscience, KNAW, Meibergdreef 47, 1105BA Amsterdam, The Netherlands; 2 Department of Psychology, University of Amsterdam, Nieuwe Achtergracht 129-B, 1018 WT Amsterdam, The Netherlands

**Keywords:** Empathy, Costly helping, Pain, Simulation theory, Mirror-pain synesthesia, Vicarious pain responders, Decision, Morality, Empathy

## Abstract

It has been argued that experiencing the pain of others motivates helping. Here, we investigate the contribution of somatic feelings while witnessing the pain of others onto costly helping decisions, by contrasting the choices and brain activity of participants that report feeling somatic feelings (self-reported mirror-pain synesthetes) against those that do not. Participants in fMRI witnessed a confederate receiving pain stimulations whose intensity they could reduce by donating money. The pain intensity could be inferred either from the facial expressions of the confederate in pain (Face condition) or from the kinematics of the pain-receiving hand (Hand condition). Our results show that self-reported mirror-pain synesthetes increase their donation more steeply, as the intensity of the observed pain increases, and their somatosensory brain activity (SII and the adjacent IPL) was more tightly associated with donation in the Hand condition. For all participants, activation in insula, SII, TPJ, pSTS, amygdala and MCC correlated with the trial by trial donation made in the Face condition, while SI and MTG activation was correlated with the donation in the Hand condition. These results further inform us about the role of somatic feelings while witnessing the pain of others in situations of costly helping**.**

## Introduction

To help someone in need we often need to sacrifice something ourselves. It has been proposed that feeling the pain of others as if it were our own is a key motivator to help. This idea was brought to prominence through Adam Smith’s theory of moral sentiments (1759): “As we have no immediate experience of what other men feel, we can form no idea of the manner in which they are affected, but by conceiving what we ourselves should feel in the like situation. Though our brother is upon the rack, […] by the imagination we place ourselves in his situation, we conceive ourselves enduring all the same torments, we enter as it were into his body, and become in some measure the same person with him, and thence form some idea of his sensations, and even feel something which, though weaker in degree, is not altogether unlike them”^1^. While other philosophers emphasize the importance of moral principles in moral decision-making, Adam Smith hereby argues that moral sentiments relate to our ability to feel something akin to the sensations of the victim: we not only act to help others because we think it wrong to let others suffer, but because we feel their pain on our own body.

In modern psychology, the notion that participants with higher levels of empathy engage in more costly helping has received some empirical support^2–4^. Given that empathy is multifaceted, what specific aspect of empathy drives prosociality is an important question^2,5^. Some have found that participants reporting more empathic concern, i.e. warm feelings for others in need, are more prosocial^3,5^. However, Adam Smith inspires us to explore a more specific question: would participants experiencing more vivid somatic pain while viewing the pain of others, be more prosocial? It is increasingly recognized that a sizable proportion of the population reports feeling tactile sensations on their own skin when observing touch on others (mirror-touch synaesthesia) or reports somatic pain in their own body while observing the pain of others (mirror-pain synesthesia/vicarious pain perception)^6–10^. Here we therefore ask whether participants reporting to experience mirror-pain synesthesia in life would help others in need more.

A valuable, albeit indirect, source of information towards this question stems from studies exploring whether individual differences in activity in primary and secondary somatosensory cortices, known to be associated with localized vicarious nociceptive signals^11^, can predict prosociality. Christov-Moore and Iacoboni^12^ found that how strongly participants activated their primary somatosensory cortex while viewing hands being poked by needles predicted generosity in later dictator games. Ma et al.^13^ similarly found that individual differences in activation of the insula and somatosensory cortices while viewing hands being poked by needles predicted later donations to charities. Finally, Gallo et al.^14^ found that trials in which participants activated their primary somatosensory cortex more strongly in response to witnessing a hand being slapped were trials in which they donated more money to reduce the slap, and that interfering with somatosensory activity using transcranial magnetic stimulation could interfere with the relationship between observed pain intensity and donations. However, these neuroscience studies do not directly address the phenomenological question of whether subjectively feeling the pain of others more somatically increases prosociality.

A secondary, and more direct approach, would be to explore whether mirror-pain synesthetes are more prosocial. What we know is that that mirror sensory synesthesia has been associated with (i) higher self-reported empathy as assessed by questionnaires for emotional reactivity^7,15^ and empathic concern^16^ (ii) enhanced empathic accuracy while recognising subtle facial expressions^15,17^ and (iii) increased self-report affect intensity when looking at emotional pictures^16^. These effects seem restricted to the affective dimension of empathy, as synesthetes do not seem to have enhanced ‘theory of mind’ cognitive empathy skills as measured by the Reading the Mind in the Eyes test^18^ and the ‘movie for the assessment of social cognition’ (MASC) test^19^. Also synesthetes seem to score lower on the social skills scale of the EQ^15,18^. Whether their increased affective empathy translates into increased prosociality however remains poorly understood. Encouraging evidence stems from Ioumpa et al.^16^ who found that mirror-sensory synesthetes donate more money to a stranger in a dictator game, but whether the added somatic sharing of pain in synesthesia would increase donations towards the individuals in pain remains unexplored.

**Figure 1:**
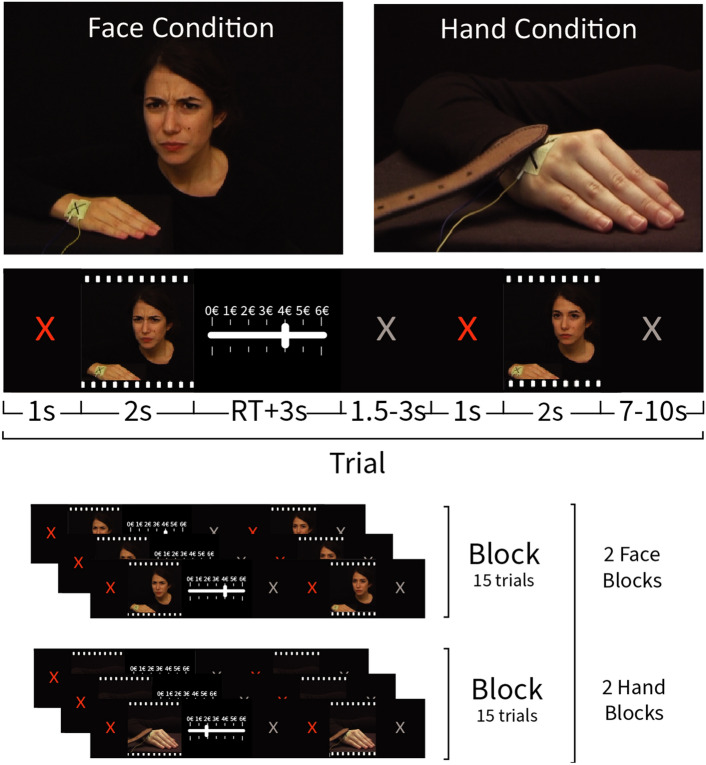
Helping task structure. Top: two screen shots taken from a video showing the confederate receiving an electrical shock on the hand and manifesting its painfulness through facial reactions and a video showing the confederate receiving a slap on her right hand. Middle: trial structure. Bottom: the experiment consisted of two Face and two Hand blocks were presented of 15 trials each.

To test this hypothesis that mirror-pain synesthetes would be willing to donate more to help alleviate the pain of others, and quantify the involvement of somatosensory cortices in prosociality, we here adapt the costly helping paradigm introduced by Gallo et al.^14^ and compare the donations of participants that do, versus those that do not, report experiencing mirror-pain. In this paradigm, participants are given the opportunity to donate money to reduce the pain of a victim they witness receive a noxious stimulation via what they believe to be a close circuit camera (Fig. 1). Importantly, the pain level varies from trial to trial, and is conveyed either by a facial expression of pain triggered by an electric shock (Face condition) or by the kinematics of a hand being slapped by a belt (Hand condition). These two stimulus types were developed to compare conditions that should only trigger vicarious distress (faces) from those that could encourage the somatosensory mapping onto a specific region of the observer's body (hand) (see Keysers et al.^11^ and Gallo et al.^14^). To decide how much money to donate, participants need to perceive the level of pain experienced by the victim in each specific trial, and tailor their donation to the specific needs of the victim in that trial—enabling us to study brain systems and individual differences in this quantitative decision-making^14^. Accordingly, if somatic mapping on the observer’s body is increased in mirror-pain synesthetes, and this motivates helping, we expect mirror-pain synesthetes to increase their donations more steeply when observing more pain particularly in the hand stimuli.

For the neural data to speak to the importance of somatic feeling in prosociality, it is important to note that the first-person experience of pain is thought to result from the combination of a sensory-discriminative dimension (where and what kind of pain do I feel?) and an affective dimension (how aversive is this pain?), with the former associated with activity in somatosensory cortices (SI and SII) while the latter is associated with activity in the anterior insula and cingulate cortex^11,20,21^. In agreement with the general notion that empathy reflects mapping the pain of others onto our own pain, brain regions and neurons involved in our own pain are activated while witnessing the pain of others^11,22–25^. That this mirroring of the affective component of pain may promote prosociality is borne out by evidence that activations in the affective pain areas correlates with helping someone in pain^3,12,13,26,27^ and inhibiting regions involved in pain experience, the cingulate in particular, reduces helping in rodents^28^. Witnessing the details of how a specific body part is harmed additionally triggers activity in SI or SII^11,12,24,29–37^ and altering activity in SI alters helping^14^. Mapping the somatic feelings reported by mirror-pain synesthetes onto this distinction would suggest that they may activate SI and SII more while witnessing the sensations and pain of others. Indeed, mirror-pain synesthetes have higher activity^38^ and increased gray matter^39^ in somatosensory cortices and anterior insula compared to controls participants; and Blakemore et al.^6^ found enhanced SI and SII activation when one mirror-touch synesthete watched movies of other people being touched compared to control participants. Hence, we might expect participants that report experiencing mirror-pain synesthesia to show more SI/SII activation when witnessing a hand being slapped, and if this somatic sharing indeed contributes to the motivation to help, this SI or SII activity should be more tightly associated with the amount of money donate to reduce that pain in mirror-pain synesthetes than non-synesthetes when witnessing the belt hitting a hand.

Additionally, recent studies have identified multivariate brain patterns that are somewhat selectively recruited when participants experience (i) physical pain^40^, (ii) the feeling of guilt^41^, and (iii) witness other people’s pain^35,42^. We might expect that while all these patterns could be associated with the motivation to help (and hence the amount of money donated in our task), a pattern trained to decode physical pain should be most tightly associated with donation in participants reporting mirror-pain synesthesia.

To shed light on the contribution of vicarious somatic pain as a motivator of helping we therefore utilize a dual approach. First, we recruited participants that report experiencing mirror-pain synesthesia and some that do not, and measured their willingness for costly helping other individuals (as in Gallo et al.^14^. By doing so, we can test our hypothesis that participants reporting to feel the pain of others somatically will help others more, and will increase their help more steeply as others experience more pain. Second, by measuring the brain activity of all participants using fMRI, we can test our hypothesis that trials in which participants have higher somatosensory activity are trials in which they decide to help more, and whether this association is tighter in self-reported synesthetes—particularly in the hand stimuli.

## Materials and methods

### Participants

In total, 32 healthy volunteers (37y ± 17SD; 32f.) with normal or corrected-to-normal vision, and no history of psychiatric, neurological, other medical problems, or any contraindication to fMRI participated in our experiment. Participants were recruited through advertisements of the experiment on social media advertising a helping decision-making study (25 participants). In addition, we also invited individuals with mirror-pain synesthesia through the contact list of participants from the study by Ioumpa et al.^16^ where they had taken part as synesthetes (7 participants).

In the end of the experiment all participants were asked to provide a “Yes–No” answer to whether they have mirror-pain synesthesia experiences during their everyday life (*“In mirror-pain synesthesia people feel on their own body the pain they observe in others. Do you have such experiences in your everyday life?”*). Those who reported having everyday mirror-pain synesthesia-like experiences were classified as self-report mirror-pain synesthetes (13 participants) and are the focus of the paper. Participants who did not report experiencing mirror-pain synesthesia experiences in their everyday life will be referred to as control participants in this study. As an additional quality check measure, participants were asked to fill the Vicarious Pain Questionnaire (VPQ) developed by Grice-Jackson et al., (2017) online after their participation in the experiment. Three participants did not fill out the VPQ. The classification revealed 7 sensory/localiser and 3 affective/general participants in our sample while the rest of our participants were classified as non-responders. Importantly, being classified as sensory/localiser was 8 times more likely amongst the self-reported mirror touch synesthetes than amongst those not self-reporting mirror touch synesthesia (Supplementary Table S1). Due to the small group sizes resulting from this finer classification, we created a responders (sensory/localiser and affective/general participants together) and no responders group, and only used this tool for exploratory behavioral analyses since using this grouping in the fMRI analyses would exclude the three participants that did not fill out the questionnaire. A more detailed description can be found in Supplementary information S1).

As two (who were recruited as synesthetes) of the 32 participants were left handed, and stimuli showed movements of the right hand of the actor, in order to reduce potential variability induced by lateralization of the brain responses, these two participants only performed the tasks off-line (i.e. no fMRI data acquired). One control fMRI participant was excluded for having a very low correlation (< 0.2) between video intensity (as given from an initial stimuli validation) and donation, for both the Hand or Face conditions. This was a criterion that we had set from the beginning for inclusion in our Helping Paradigm analyses. Thus we ended up with fMRI data for 29 participants (11 self report mirror-pain synesthetes and 18 control participants) and behavioral data for 31 participants (13 self report mirror-pain synesthetes and 18 control participants). All experimental protocols were approved by the Ethics Committee of the University of Amsterdam, The Netherlands (2017-EXT-8201). Informed consent was obtained from all subjects and authorization for the publication of images. All methods were carried out by relevant guidelines and regulations.

### fMRI helping paradigm

#### Stimuli

The same set of stimuli as in Gallo et al.^14^ was used. Two types of videos were presented. One showing the confederate receiving an electroshock on the hand and expressing the pain she felt by only reacting with facial expression. No hand movement or muscle twitch was visible in these videos (Face videos). The other showed a belt hitting the dorsum of the confederate right hand, and the confederate expressing how much pain she felt by a reaction of the hand alone (Hand videos). The face was not visible in the latter stimuli. Electrodes were attached to the skin of the confederate with tape, which was also retained during the Hand video recording. A black ‘X’ was drawn on the tape marking the electrode's position. Each stimulation had a duration of 1000 ms and varied in intensity ranging from 0.2 to 8.0 mA. All videos lasted 2 s and were neutral during the first second. The Face videos started with the face in a neutral expression that was kept neutral until the stimulation. The Hand videos started with the belt laying on the hand dorsum until the end of the first second when it was lifted in order to hit.

Stimuli were validated by an independent group of subjects (45 volunteers, 32.36y ± 10.36SD; 23f.), who were asked to watch 331 videos and rate the intensity of the pain displayed on a scale from 1 to 10, with ‘1’ being ‘just a simple touch sensation’ and ‘10’ being ‘most intense imaginable pain’. The Face and Hand videos were presented in separate blocks and were counterbalanced across participants. Participants were informed that videos with intensity higher than 6 would never be delivered so that the pain stimulations would be painful but bearable. For the first video of each trial, we selected 60 videos (30 for the Face and 30 for the Hand condition) with ratings from 1 to 6 with matching intensity for the Face and Hand conditions (mean ± SD, 4 ± 1.558). Each one of these videos was shown once. For the second video of each trial, we selected 140 videos (70 for the Face and 70 for the Hand condition) with matching intensity for the Face and Hand conditions (mean ± SD, 3.171 ± 1.200). Since the intensity of the second video depended on participants’ donation, different participants saw a different selection of these videos. Videos were edited using Adobe Premiere Pro CS6 (Adobe, San Jose, CA, USA).

#### Task

The task was an fMRI adaptation of the Helping task as published in Gallo et al.^14^. Participants performed 60 trials in which they watched a first (pre-recorded) video of the confederate receiving a painful stimulation. The intensity of the stimulation could vary between 1 and 6 on a 10 point pain scale, and was chosen on each trial randomly by the computer program. In each trial participants received 6 euro credits, and could decide to donate some of them in order to reduce the intensity of the second stimulation to the confederate. Each donated credit reduced the next stimulation by 1 point on the 10 point pain scale. Participants then watched a second video showing the confederate’s response to the second stimulation. At the end of the task, participants were paid the sum of the amount of money that they had kept for themselves from all the trials divided by 10. Prosocial behavior was captured as the average number of credits given up in all trials (“donation”). Hand and Face videos were presented in separate sessions that were randomized across participants. In total there were 2 sessions of 15 trials for the face and hand videos.

We used the same cover story used in Gallo et al.^14^. Each participant was paired with what they believed to be another participant like them, although in reality it was a confederate. They drew lots to decide who plays the role of the decision maker and of the pain-receiver. The lots were rigged so that the confederate would always be the pain-receiver. The participant was then taken to the scanning room while the confederate was brought to an adjacent room, with a fake filming set up. Participants were misled to believe that the pain stimulations were delivered to the confederate and displayed to them in fMRI in real-time while in reality pre-recorded videos were used. All participants were presented the same set of videos in a randomized order.

At the end of the fMRI tasks, participants were debriefed. To assess whether they believed the cover story, they were asked to answer the question ‘Do you think the experimental setup was realistic enough to believe it’ on a scale from 1 (strongly disagree) to 7 (strongly agree) in an exit questionnaire. All participants reported that they at least somewhat agreed with the statement (i.e. 5 or higher). Participants were also asked to fill out the interpersonal reactivity index (IRI) empathy questionnaire^43^, and the money attitude scale (MAS)^44^.

The task was programmed in Presentation (www.neurobs.com), and presented under Windows 10 on a 32 inch BOLD screen from Cambridge Research Systems visible to participants through a mirror (distance eye to mirror: ~ 10 cm; from mirror to the screen: ~ 148 cm). The timing of the task was adapted to the requirements of fMRI: Each trial started with a jittered gray fixation cross lasting 7–10 s (Fig. 1). Then a red fixation cross appeared for 1 s, followed by the first video presentation and the donation scale. Participants could make their choice without a time restriction. In order to make their choice they could move the bar in the scale using their right index and middle finger. After 3 s of inactivity the system would automatically register their response. Then a jittered gray fixation cross lasting 1.5–3 s would follow, then a 1 s red cross and the second video. The role of the red fixation crosses was to capture participants’ attention just before a video appears.

#### Analysis of behavioral data

Statistical analyses were performed using JASP (https://jasp-stats.org, version 0.11.1), to provide both Bayes factors and *p* values. Bayes factors allowed us to differentiate between evidence of absence and evidence of the presence of an effect, and therefore complemented traditional frequentist statistics as *p*-values cannot quantify evidence for the absence of an effect^45^. We used traditional bounds of BF_10_ > 3 to infer the presence of an effect and BF_10_ < 1/3 to infer the absence of an effect^45^. We report correlations by indicating Pearson Correlation Coefficients (r), Bayes factors and *p* values. Two-tailed tests are indicated by BF_10_, i.e. *p*(Data|H_1_)/*p*(Data|H_0_) while one-tailed tests are indicated by BF_+0_. Where ANOVAs were used, we report BF_incl_ which reports the probability of the data given a model including the factor divided by the average probability of the data given the models not including that factor. When using t-tests, we examined normality using the Shapiro–Wilk’s test. If normality was preserved, we report t tests and t-values and if not, we report Wilcoxon signed rank tests, as indicated by W values. We always used default priors for Bayesian statistics as used in JASP. The relationship between the intensity of video1 and the donations was analyzed using a random intercept linear mixed model considering the video type (Hand vs Face) as a factor and subject as random effect.

#### MRI data acquisition

MRI images were acquired with a 3-Tesla Philips Ingenia CX system using a 32-channel head coil. One T1-weighted structural image (matrix = 240 × 222; 170 slices; voxel size = 1 × 1x1mm) was collected per participant together with an average of 775.83 EPI volumes ± 23.11 SD (matrix M × P: 80 × 78; 32 transversal slices acquired in ascending order; TR = 1.7 s; TE = 27.6 ms; flip angle: 72.90°; voxel size = 3 × 3 × 3 mm, including a 0.349 mm slice gap).

#### fMRI data preprocessing

The MRI data were processed in SPM12. EPI images were slice-time corrected to the middle slice and realigned to the mean EPI. High quality T1 images were coregistered to the mean EPI image and segmented. The normalization parameters computed during the segmentation were used to normalize the gray matter segment (1 mm × 1 mm × 1 mm) and the EPIs (2 mm × 2 mm × 2 mm) to the MNI templates. In the end, EPIs images were smoothed with a 6 mm kernel.

***fMRI data analyses.***

Our neural analyses aimed at testing whether the association between donations and brain activity while witnessing the pain of others involves somatosensory cortices, particularly in the Hand condition, and whether this association is stronger in participants reporting mirror pain-synesthesia compared to those who did not.

#### GLM analyses

Our analyses focused on how brain activity during the first video related to donation. The second videos were modeled, but as a variable of no interest as they were the result of a decision rather than the cause of that decision. For each of our four sessions (two that presented the Face stimuli and two the Hand), our fMRI design matrix included the following regressors. (1) A first video regressor that started with a red fixation cross and ended with the end of the first video. We call this regressor the main effect of Face or Hand Video1. The red cross was included in this regressor as it was always presented at a fixed time interval of 1 s before the first video and separating their contribution to the BOLD signal would not have been possible. (2) This regressor had the donation made in each trial as a parametric modulator, creating a FaceDonation and a HandDonation parametric modulator. The donation values for each run were standardized with the zscore function of MATLAB before being inserted as a regressor. Standardization here was used so that the parameter estimate of the parametric modulator becomes independent of a participants range of donation, and reflects how tightly brain activity is associated with donation (in the sense of a correlation) rather than the specific slope. (3) A decision regressor started with the appearance of the donation scale and ended 3 s after the last button press of the participant, when the scale disappeared. (4) The second video regressor was aligned with the presentation of the red cross before the second video and ended with the end of the second video. (5) A regressor with the standardized donation made in this trial as a parametric modulator for video 2. (6–11) Finally, 6 regressors of no interest were included to model head translations and rotations.

We then brought the parameter estimate images for FaceDonation, HandDonation and Face and Hand main effects into four separate t-tests and contrasted them against zero. Two sample t-tests were instead used to compare activity across groups. Results were thresholded at *p*_unc_ < 0.001 and 5% family-wised error (FWE) corrected at the cluster level by setting the minimum cluster-size k to the FEWc value calculated by SPM after visualizing the results at *p*_unc_ < 0.001 k = 10 (Eklund et al., 2016).

#### Neurological signatures analyses

Because of the difficulties to associate changes in brain activity in a single location with specific mental processes without facing reverse inference issues^46^, we additionally used three multivariate signatures. These maps quite selectively detect whether participants perceive other people's pain (vicarious pain signature, VPS)^35^, feel their own pain (neurological pain signature, NPS)^40^ or feel interpersonal guilt^41^. In order to explore if signals in these networks covaried with FaceDonation and HandDonation we brought the signatures into our fMRI analysis space using ImageCalc, extracted the FaceDonation and HandDonation parameter estimate image (β_FaceDonation_ and β_HandDonation_) from each participant and dot-multiplied them separately with the three signatures. The result indicated how much the covariance with FaceDonation and HandDonation loads on the VPS, NPS and guilt signatures. We then brought these values into JASP, and compared them against zero and checked for differences between individuals reporting mirror-pain synesthesia experiences and those who did not.

## Results

### Behavioral results

Participants donated the same amount on average for the face (mean ± SD, 2.538 ± 1.066) and hand (mean ± SD, 2.509 ± 1.149) conditions (t_(30)_ = 0.289, *p* = 0.774, Cohen’s d = 0.052, BF_10_ = 0.199). Also participants donated more money on trials in which the confederate expressed more pain both for the Face (average correlation across participants r = 0.816, SD = 0.168, *p* = 2 × 10^–4^, BF_10_ = 162 and the Hand (average correlation across participants r = 0.634, SD = 0.220, *p* = 0.01, BF_10_ = 6) conditions.

Our initial hypothesis was that self-declared mirror-pain synesthetes would behave more prosocially and increase their donations more steeply when observing more pain, particularly in the hand stimuli.

**Figure 2:**
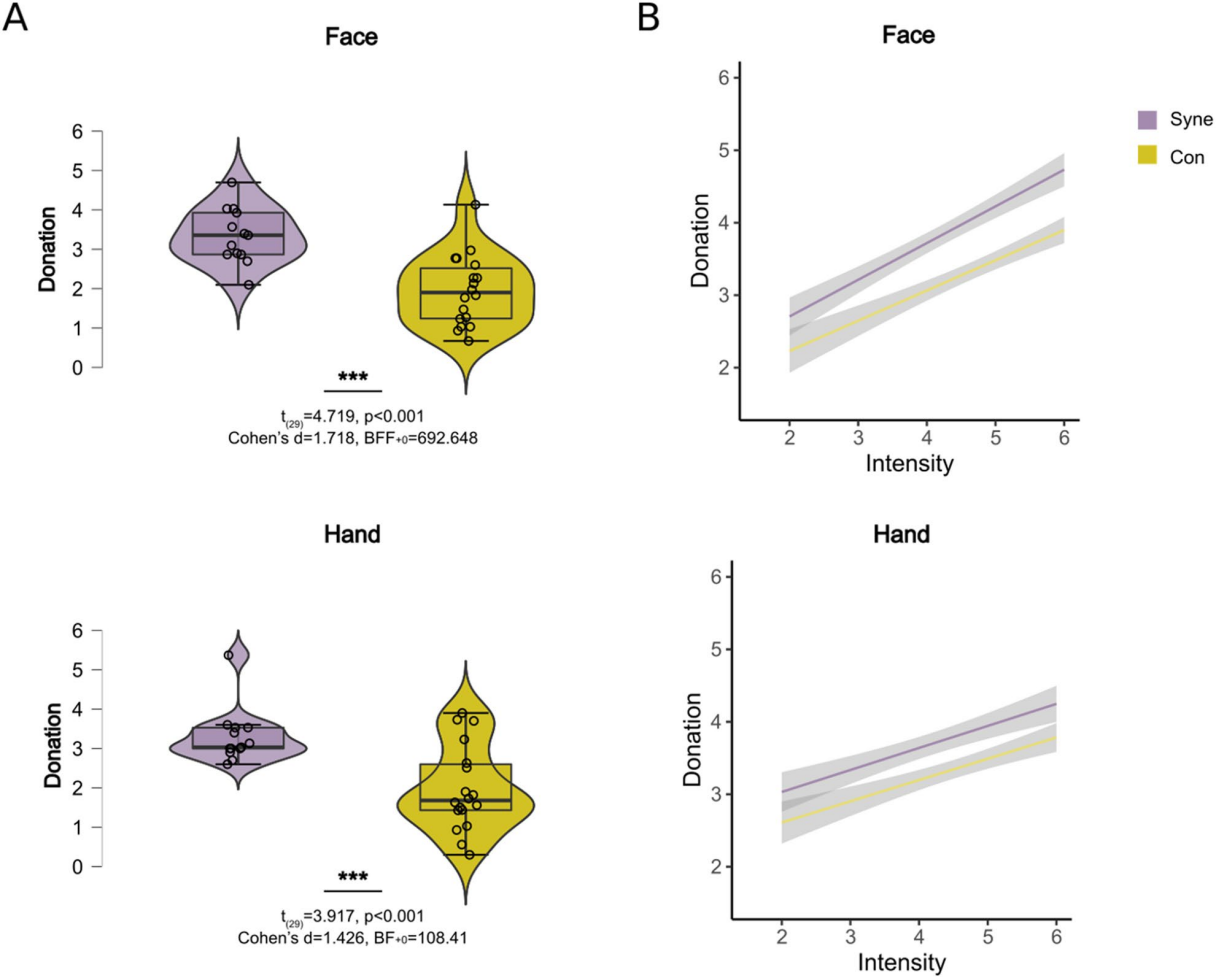
(**A**) Average of donation for the Face and Hand conditions for self-report synesthetes (cyan) and controls (yellow). Violin plots represent the distribution, the box-plot within, the median, and the whisker the quartiles. The BF_10_ and *p*-values between the violin plots represent the results of the comparison between individuals reporting mirror-pain synesthesia experiences and those who did not. (**B**) Correlation between Donation and Intensity for the Face and Hand conditions for self-report synesthetes and controls.

Figure 2 shows that synesthetes donated more money in order to help than control participants for the Face (t_(29)_ = 4.719, *p* < 0.001, Cohen’s d = 1.718, BF_+0_ = 692.648) and Hand conditions (t_(29)_ = 3.917, *p* < 0.001, Cohen’s d = 1.426, BF_+0_ = 108.411). We analyzed the relationship between the intensity of video1 (as resulted from the stimuli validation described in Gallo et al.^14^) and the donations as a function of the stimulus (Hand vs Face) and self-report synesthesia using a random intercept linear mixed model with subject as random effect. Decomposing the effect of these factors revealed that participants gave 2.54 euros per trial on average, and that the donation depended strongly on the intensity of video1 (F_(1,1764)_ = 1369.176, *p* < 0.001), with a slope of 0.65. Importantly, self-declared synesthetes gave more on average (3.32 ± 0.66 SD) than controls (1.94 ± 0.94 SD) and also had a steeper slope (F_(1,1764)_ = 23.354, *p* < 0.001), i.e. adapted their donations more to the intensity of the victims pain. This group difference did not depend on whether Face or Hand stimuli were seen (F_(1,1764)_ = 0.113, *p* = 0.736).

Comparing responders and non-responder groups from the VPQ we observed the same tendency, with responders donating significantly more for the face (t_(26)_ = − 1.722, *p* = 0.048, Cohen’s d = − 0.679, BF_+0_ = 1.951) and hand (t_(26)_ = − 1.877, *p* = 0.036, Cohen’s d = − 0.740, BF_+0_ = 2.406) conditions when using frequentist statistics, while the Bayesian statistics were less conclusive, although showing a similar trend.

None of the subscales of IRI or MAS correlated with the donation and the Bayesian statistics were close to evidence for absence of a correlation (all r < 0.214, all *p* > 0.247, all BF_10_ < 0.424, Supplementary Table S2). Additionally, there was no support for differences in empathy between the synesthetes and control for none of the IRI subscales (independent sample t-test, FS: t = − 0.566, BF_10_ = 0.433; PT: t = − 0.817, BF_10_ = 0.49; EC: t = − 0.566, BF_10_ = 0.432; PD: t = − 0.353, BF_10_ = 0.404). Further analyses show that the differences in donation between the two groups were not explained by our measures of trait empathy (Supplementary Table S2).

### fMRI results

#### GLM analyses

Comparing the main effect of Face and Hand during the the first video revealed significant differences across these two types of stimuli: the IFG and IPL showed higher BOLD signal for Face than Hand stimuli and SII, insula and the calcarine gyrus showed higher BOLD signal for the Hand than Face (Supplementary Figure S2 and Supplementary Table S4).

**Figure 3:**
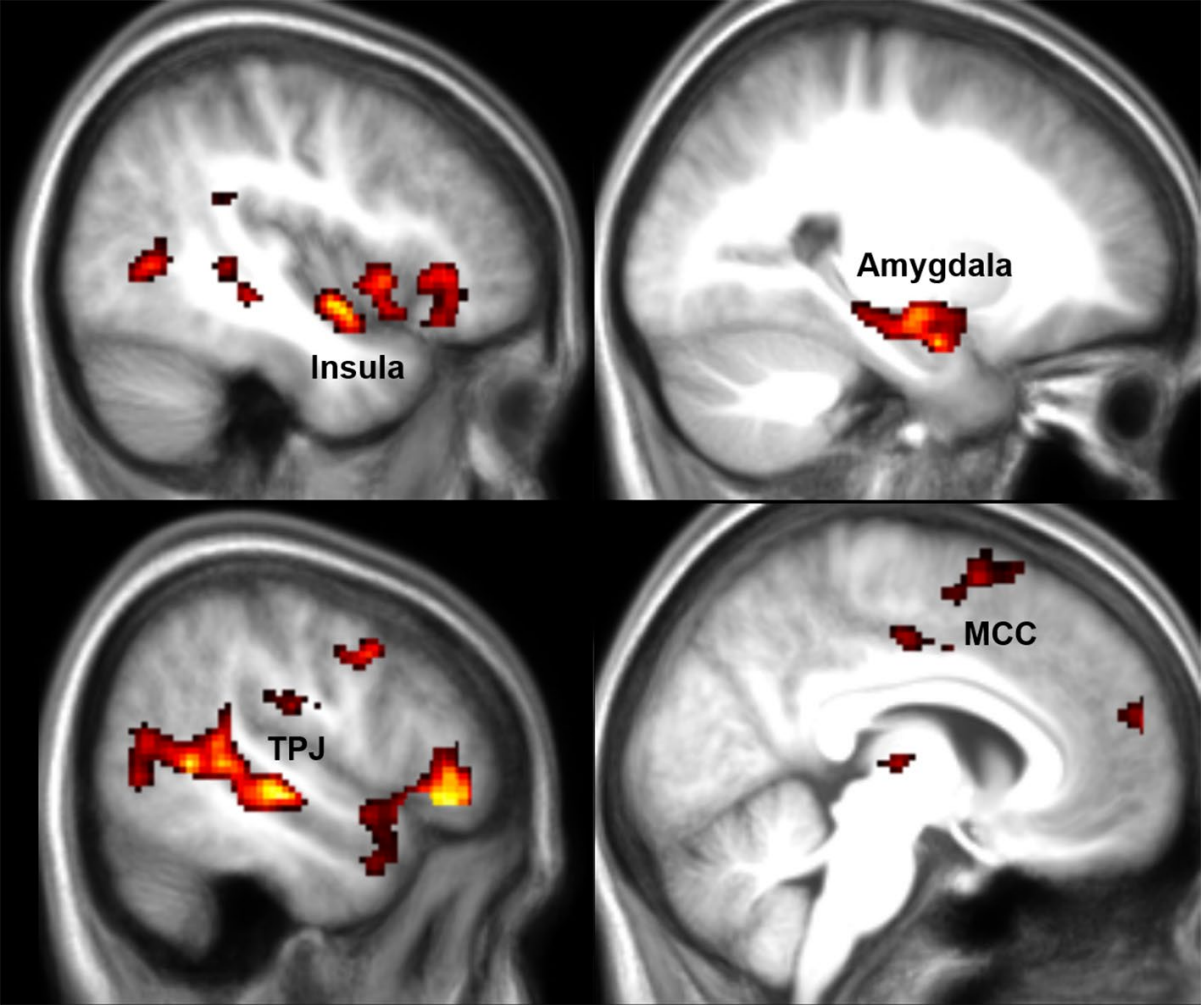
fMRI results for Face Donation. Results of a linear regression on the parametric modulator for the first video and trial-by-trial donation in the Face condition. Results are FWE cluster-corrected at *p* < 0.05 (*p*_unc_ < 0.001, k = FWEc = 110 voxels, 3.34 < t < 8). This identifies voxels with signals that increase for higher donation.

To identify whether the circuitry involved in donations differs based on how the pain of others is conveyed (through facial expressions or movements of the hand) we localized voxels in which activity correlated with Donation in all participants for Hand and Face trials separately. For the Face condition, we observed that the more money participants donated the higher the BOLD signal in the insula, SII, TPJ, pSTS, amygdala and MCC (Fig. 3 and Table 1).

**Table 1:** Results of the voxelwise analysis.

Cluster size	# Voxels in cyto	% Cluster	Hem	Cyto or anatomical description	% Area	Peak Information			
						T	x	y	z
FaceDonation All Participants p_unc_ < 0.001 k = FWEc = 110									
3656	137.8	3.8	R	Area 45 IFG (p. Triangularis)	13.3	8.38	48	30	− 2
			R	Area 45 IFG (p. Orbitalis)		7.77	50	28	− 6
			R	Area 45 IFG (p. Orbitalis)		7.19	52	32	− 8
	135.1	3.7	R	Area PFcm (IPL)	41.5				
	104.8	2.9	R	Area PF (IPL)	15.5				
	92.9	2.5	R	Area TE 3	8.9				
	59.5	1.6	R	Amygdala (LB)	27.9				
	44.1	1.2	R	Area hOc5 [V5/MT]	75.8				
	42.3	1.2	R	Area OP1 [SII]	10.8				
	39.8	1.1	R	Area Id1 Insula	24.3	7.74	40	− 4	− 10
	36	1	R	Area PFm (IPL)	5.1				
	29.8	0.8	R	Area PFop (IPL)	13				
	25.3	0.7	R	Area hOc4la	2.8				
	24.6	0.7	R	Amygdala (CM)	90				
	18	0.5	R	CA2 (Hippocampus)	29.9				
	17.8	0.5	R	CA3 (Hippocampus)	30				
	16.5	0.5	R	Area PGp (IPL)	1.7				
	15.6	0.4	R	Amygdala (SF)	32.8				
	15.3	0.4	R	CA1 (Hippocampus)	5.3				
	15.1	0.4	R	BF (Ch 4)	36.3				
	13.5	0.4	R	Area PGa (IPL)	1.8				
	8.8	0.2	R	Subiculum	2.3				
	7.9	0.2	R	HATA Region	36				
	4.3	0.1	R	Amygdala (AStr)	27.6				
	3	0.1	R	Thal: Parietal	0.9				
	2.9	0.1	R	Area Fo2	0.3				
	2	0.1	R	DG (Hippocampus)	1.6				
	2	0.1	R	Area 44	0.3				
	1.1	0	R	Area 3a	0.6				
	1	0	R	Area 3b	0.2				
			R	Middle Temporal Gyrus		8.61	46	− 38	4
			R	Superior Temporal Gyrus		7.78	50	− 24	− 6
			R	Insula		6.23	38	8	− 8
			R	Hippocampus		5.97	22	− 6	− 16
2496	152.8	6.1	L	Thal: Prefrontal	24.2				
	83.6	3.4	L	Area Id1	71.2				
	57	2.3	L	Amygdala (LB)	23.5	6.46	− 24	− 6	− 22
	46.1	1.8	L	Area 45	6.6				
	43.5	1.7	L	Subiculum	11.4				
	39.3	1.6	L	Amygdala (CM)	90.8	7.29	− 22	− 12	− 18
	33	1.3	L	DG (Hippocampus)	20.1	6.03	− 28	− 24	− 16
	26.6	1.1	L	CA3 (Hippocampus)	65.9				
	26.6	1.1	L	Area 44	3				
	26.3	1.1	R	Thal: Prefrontal	4.7				
	25	1	L	Thal: Premotor	21.1				
	16	0.6	L	BF (Ch 4)	31.9				
	15.8	0.6	L	Thal: Parietal	5				
	10.5	0.4	L	CA2 (Hippocampus)	19.7				
	10.3	0.4	L	Thal: Motor	20.7				
	8.8	0.4	L	CA1 (Hippocampus)	4				
	7.9	0.3	L	Area TE 3	0.9				
	7.8	0.3	L	Amygdala (AStr)	36				
	7.4	0.3	L	HATA Region	31.6				
	4.5	0.2	L	Thal: Somatosensory	14.5				
	3.3	0.1	L	Area Fo2 Insula	0.3	6.94	− 30	14	− 18
	2.9	0.1	L	Amygdala (SF)	7.9				
	1.9	0.1	L	Thal: Visual	2.1				
	0.9	0	R	Thal: Temporal	0.2				
	0.5	0	L	Area TE 1.2	0.4				
			L	Temporal Pole		6.91	− 40	2	− 18
			L	Insula		6.88	− 42	8	− 4
			L	IFG (p. Orbitalis)		6.42	− 44	26	− 6
			L	IFG (p. Triangularis)		6.37	− 40	28	− 2
			L	Amygdala		5.81	− 30	2	− 18
517	35.5	6.9	L	Area PFcm (IPL) SupraMarginal Gyrus	11	4.40	− 54	− 40	28
	34.1	6.6	L	Area PF (IPL) Superior Temporal Gyrus	6.5	4.76	− 64	− 48	14
			L	Area PF (IPL) Superior Temporal Gyrus		3.99	− 62	− 42	24
	28	5.4	L	Area PGa (IPL) Middle Temporal Gyrus	4.4	5.45	− 56	− 50	22
	27.6	5.3	L	Area PFm (IPL) Middle Temporal Gyrus	4.8	4.55	− 62	− 52	12
	10.8	2.1	L	Area PGp (IPL) Middle Temporal Gyrus	1.3	3.91	− 52	− 70	16
	2.6	0.5	L	Area TE 3 Middle Temporal Gyrus	0.3	4.91	− 62	− 38	8
	1	0.2	L	Area PFop (IPL)	0.5				
	0.6	0.1	L	Area hOc4la	0.1				
	0.1	0	L	Area hOc5 [V5/MT]	0.2				
	0.1	0	L	Area PFt (IPL)	0				
320	21.9	6.8	L	Area Fp2 Superior Medial Gyrus	3	4.12	− 6	60	14
	13	4.1	R	Area Fp2 Superior Medial Gyrus	2.1	4.65	4	58	18
	0.1	0	L	Area Fp1	0				
238			L	MCC		5.93	− 8	− 18	42
			R	MCC		4.53	2	− 12	42
			R	MCC		4.37	− 16	44	6
223			R	Posterior-Medial Frontal		4.59	6	6	62
110			R	Precentral Gyrus		5.37	52	6	38
HandDonation All Participants p_unc_ < 0.001 k = FWEc = 122									
122			R	Medial Temporal Pole		48	8	− 24	48
			R	Temporal Pole		46	14	− 24	46
			R	Middle Temporal Gyrus		52	2	− 30	52
HandDonation Synesthetes-Controls p_unc_ < 0.001 k = FWEc = 379									
379	87.5	23.1	L	Area PFop (IPL) SupraMarginal Gyrus	39.4	5.13	− 62	− 26	24
			L	Area PFop (IPL) Superior Temporal Gyrus		5.10	− 64	− 28	22
	71.3	18.8	L	Area PFcm (IPL) Superior Temporal Gyrus	22	5.07	− 52	− 32	18
	56.4	14.9	L	Area OP1 [SII]	15.1				
	55.6	14.7	L	Area OP4 [PV] Postcentral Gyrus DDffcccpPostcentral Gyrus Postcentral Gyrus Postcentral Gyrus Postcentral Gyrus	15.4	5.55	− 64	− 16	16
			L	Area OP4 [PV] Superior Temporal Gyrus		3.55	− 54	− 14	10
	15.6	4.1	L	Area PF (IPL) SupraMarginal Gyrus	3	3.89	− 64	− 38	26
	13	3.4	L	Area TE 3	1.5				
	4	1.1	L	Area PFt (IPL)	0.7				
	3.8	1	L	Area OP3 [VS]	2.7				
	1.3	0.3	L	Area TE 1.0	1				

Brain activations for the FaceDonation for all participants together, for the HandDonation for all participants and for the HandDonation for Synesthetes-Control participants. Regions were labeled using SPM Anatomy Toolbox. From left to right: the cluster size in number of voxels, the number of voxels falling in a cyto-architectonic area, the percentage of the cluster that falls in the cyto-architectonic area, the hemisphere (L = left; R = right), the name of the cyto-architectonic area when available or the anatomical description, the percentage of the area that is activated by the cluster, the t values of the peaks associated with the cluster followed by their MNI coordinates in mm.

**Figure 4:**
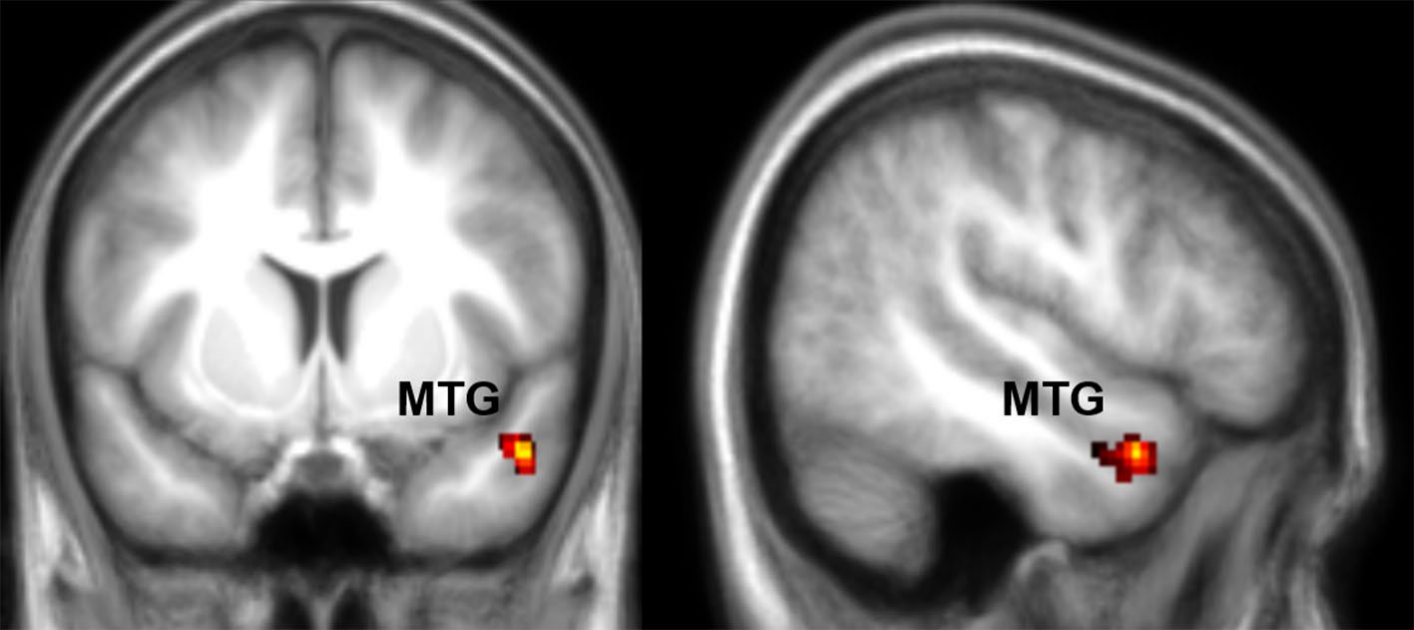
fMRI results for Hand Donation. Results of a linear regression on the parametric modulator for the first video and trial-by-trial donation in the Hand condition. Results are FWE cluster-corrected at *p* < 0.05 (*p*_unc_ < 0.001, k = FWEc = 122 voxels, 3.34 < t < 5). This identifies voxels with signals that increase for higher donation.

For Hand trials, we observed that the more money participants donated the higher the BOLD signal in the Middle Temporal Gyrus (Fig. 4 and Table 1). Based on EEG results in Gallo et al.^14^ we expected SI to also predict donation in the Hand condition. We then looked at the results at uncorrected *p*_unc_ = 0.01 and found activation in SII, insula, ACC, MMC among others (Supplementary Figure S3 and Supplementary Table S5), but significant voxels in SI remained conspicuously rare. Given our sample size, this however does not exclude the existence of small to medium effect sizes in SI, as a power-analysis with 29 participants, a power of 80% and voxelwise thresholds of *p* = 0.001 or *p* = 0.01 indicates that we would be sufficiently powered only to find strong (d = 0.8) or medium (d = 0.6) effect sizes with 80% likelihood. Driven by the surprising lack of findings for SI we therefore supplemented the univariate approach with a multivariate approach (partial least-square regression) which sometimes has higher sensitivity than univariate analyses for specific regions of interest, to further explore the role of SI in the Hand condition (procedure described in Supplementary information S3). This multivariate approach revealed that SI does indeed contain information that relates to the magnitude of donations when a pain is conveyed by the hand (Supplementary Figure S4).

To explore the difference between the association with donation for Face and Hand trials in more detail, we directly compared the parameter estimates for the parametric donation modulators for Hand and Face using a paired sample t-test, but no significant results survived for either direction (t = 3.34, *p*_unc_ < 0.001). This could suggest that a similar network is activated during the Hand condition as well but less strongly or in a more variable way across participants.

**Figure 5:**
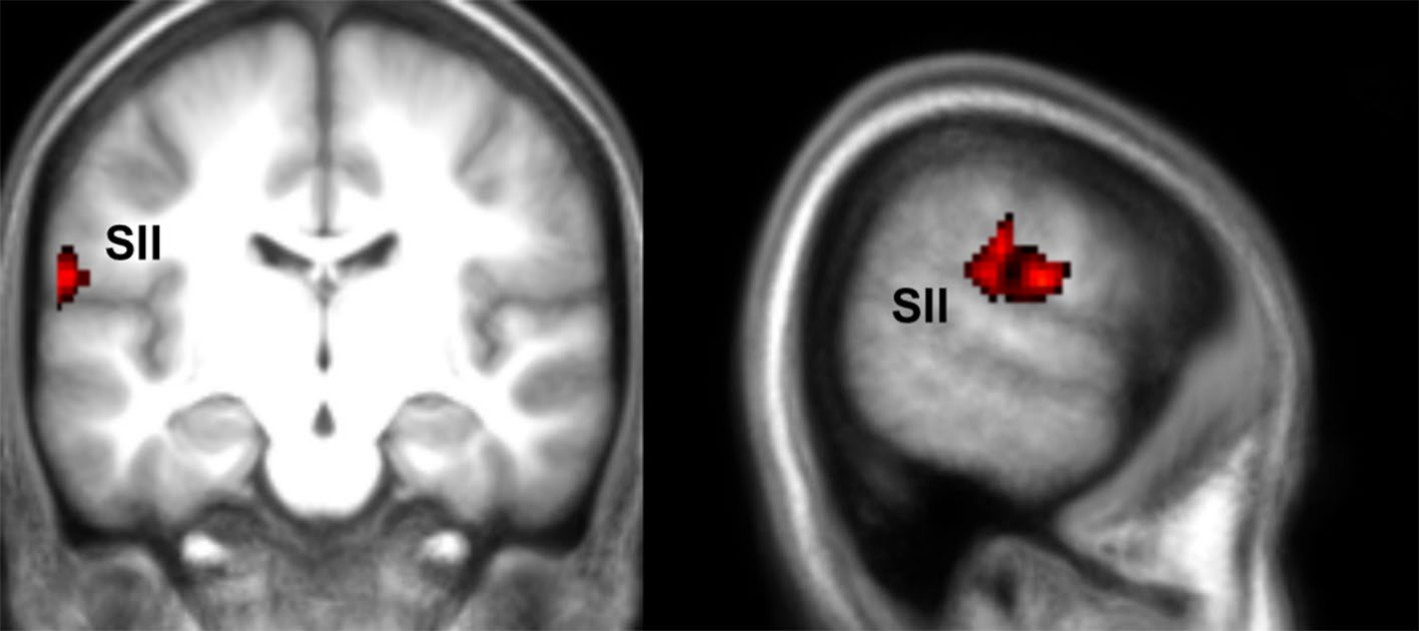
fMRI results for Hand Donation group comparison. Results of a two sample t-test between the self-report synesthetes and control participants for the HandDonation. Results are FWE cluster-corrected at *p* < 0.05 (*p*_unc_ < 0.001, k = FWEc = 379, 3.34 < t < 8). This identifies voxels with signals that increase for higher donation. The reverse contrasts did not yield results.

To explore whether self-report mirror-pain synesthetes differed in their brain activations compared to self-report non-synesthetes, we performed a two sample t-test comparing the FaceDonation and HandDonation parametric modulators across these two groups. We observed higher parameter estimates in SII and the adjacent parietal operculum for the self-report synesthetes compared to the non-synesthetes for the HandDonation regressor contrast (Fig. 5 and Table 1). This suggests that, as hypothesized, donations are more tightly associated with somatosensory activity in synesthetes than controls. The FaceDonation comparison did not reveal significant differences.

Analysis on the main effect of video1 independently of donation can be found in the supplementary material (Supplementary Fig. S1 and Supplementary Table S3). As expected the videos elicited activity in an extensive network that included, but was not limited to, visual cortices and regions often responding to the pain of others, such as the insular and cingulate cortices. Results from the direct comparisons between the main effect of Face and Hand for video 1 can also be found in the supplementary material (Supplementary Fig. S1 and Supplementary Table S3).

#### Neurological signatures analyses

When looking at the result of the signature analyses, through one sample t-tests against zero, we found evidence for a loading of the FaceDonation condition for NPS (W = 343, *p* = 0.003, BF_+0_ = 12.975), for VPS (W = 362, *p* < 0.001, BF_+0_ = 242.022) and also for the guilt signature (t_(28)_ = 2.289, *p* = 0.015, Cohen’s d = 0.425, BF_+0_ = 3.604). The same was the case for the HandDonation condition for NPS (t_(28)_ = 2.010, *p* = 0.027, Cohen’s d = 0.373, BF_+0_ = 2.203), VPS (t_(28)_ = 1.795, *p* = 0.042, Cohen’s d = 0.333, BF_+0_ = 1.548) and the guilt signature (t_(28)_ = 3.581, *p* < 0.001 Cohen’s d = 0.665, BF_+0_ = 53.887). These results show that both the pain observation network (VPS) and physical pain network (NPS) of our participants are more active on trials in which participants donated more. The same seems to be the case for the guilt network. For the NPS and VPS for the FaceDonation a Shapiro–Wilk’s test rejected the null-hypothesis of a normal distribution, thus a non-parametric Wilcoxon signed rank test vs zero was used.

**Figure 6:**
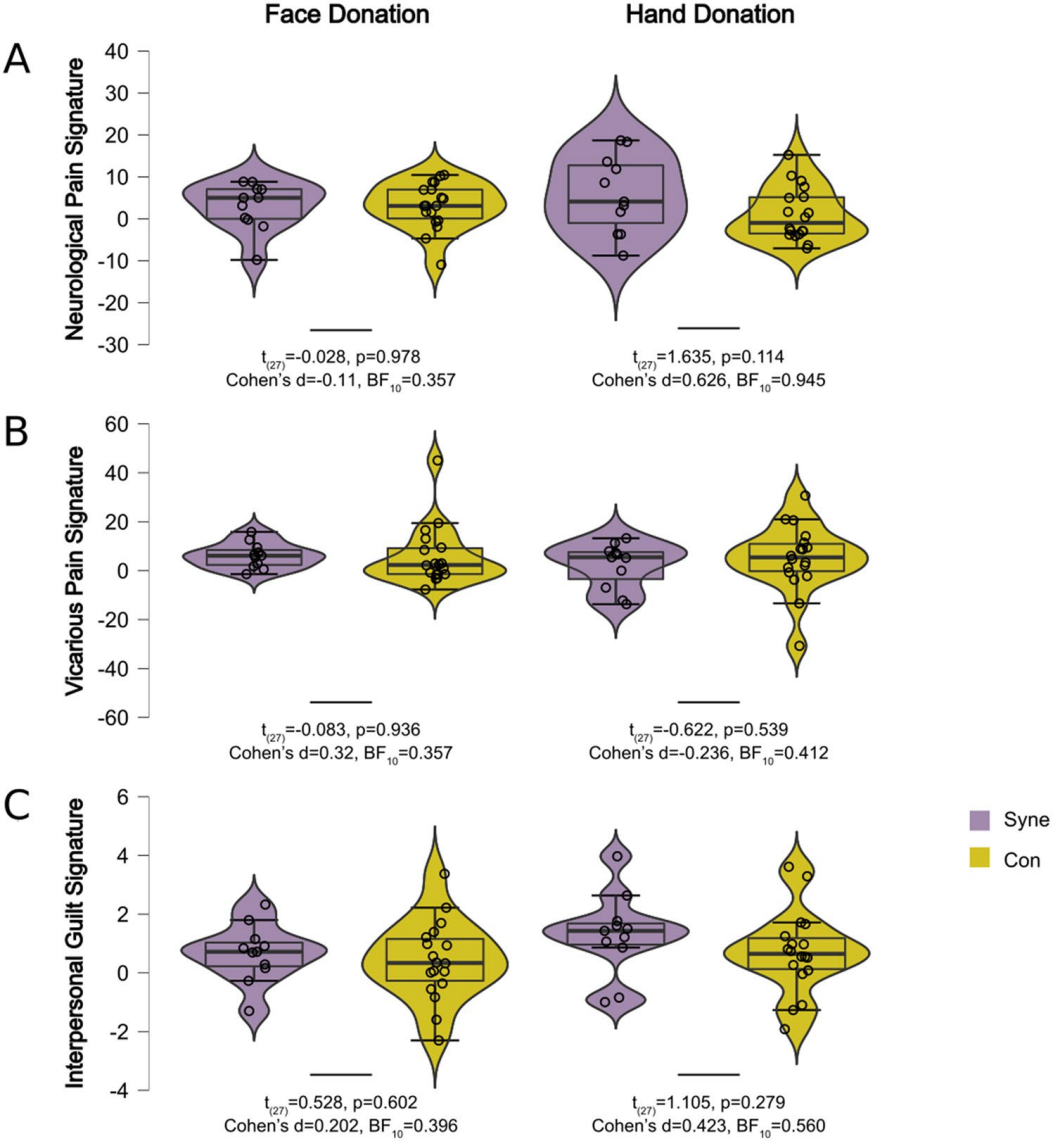
(**A**) Results of the Neurological Pain Signature analyses for the FaceDonation and HandDonation. (**B**) Results of the Vicarious Pain Signature analyses for the FaceDonation and HandDonation. (**C**) Results of the Interpersonal Guilt Signature analyses for the FaceDonation and HandDonation. All results are displayed in arbitrary units. Violin plots represent the distribution, the box-plot within the median and the whisker the quartiles. The BF_10_ and *p*-values between the violin plots represent the results of the comparison between individuals reporting mirror-pain synesthesia (cyan) experiences and those who did not (yellow).

Comparing the loadings on the three signatures of the FaceDonation and HandDonation between individuals reporting mirror-pain synesthesia experiences and those who did not, did not show any significant differences and there seemed to be evidence of absence of a difference. Comparison between self-report mirror-pain synesthetes vs controls for FaceDonation: NPS (t_(27)_ = − 0.028, *p* = 0.978, Cohen’s d = − 0.11, BF_10_ = 0.357), VPS (t_(27)_ = 0.083, *p* = 0.934, Cohen’s d = 0.032, BF_10_ = 0.357) and for the guilt signature (t_(27)_ = 0.528, *p* = 0.602, Cohen’s d = 0.202, BF_10_ = 0.396). For HandDonation: NPS (t_(27)_ = 1.635, *p* = 0.114, Cohen’s d = 0.626, BF_10_ = 0.945), VPS (t_(27)_ = − 0.622, *p* = 0.539, Cohen’s d = − 0.236, BF_10_ = 0.412) and the guilt signature (t_(27)_ = 1.105, *p* = 0.279, Cohen’s d = 0.423, BF_10_ = 0.560) (Fig. 6).

## Discussion

To shed light onto the contribution of somatic feelings on costly helping, we contrasted the choices and brain activity of participants that report feeling such somatic feelings (self-reported mirror-pain synesthetes) against those that do not. Our results showed enhanced prosocial behavior and enhanced association of decision-making with somatosensory brain activity, in the Hand condition, in self-reported mirror-pain synesthetes.

Behaviorally, in line with the notion that somatic feelings may contribute to a motivation to help, individuals reporting mirror-pain synesthesia donated more money to reduce the pain of another individual, and their donations increased more steeply as the witnessed pain became more intense. Somewhat surprisingly, this was true whether the pain was perceived from the Face or Hand, although we had expected this to be more strongly the case for the Hand stimuli as they were designed to invite viewers to mirror their observation more specifically on their own hand. However it should be noted that in our Face stimuli, even if the pain intensity was conveyed by the facial expressions of the confederate, the hand receiving the electrical shock was also visible. Thus we cannot rule out that mirroring on participants' hands still occurred albeit to a lesser extent. That self-declared synesthetes donated more money than control participants independently of the details of the stimuli dovetails with prior findings that mirror-pain synesthetes donate more money than control participants in anonymous dictator’s games^16^ and self-report higher levels of emotional reactivity in the Empathy Quotient^7^. A history of sharing the sensations of others more physically may thus lead to a general increase of willingness to help in mirror-pain synesthetes, even if the specific stimuli presented at the time of decision-making are not optimized to trigger mirror-pain sensations (like the Face stimuli). Notably, self-reported synesthesia, but not individual differences in trait empathy, was associated with differences in donation in our paradigm, providing some support for the notion of Adam Smith that somatosensory feelings akin to those of the victim represent a significant contribution to moral sentiments and the motivation to help.

One might have hypothesized that cognitive aspects of empathy could account for reports of mirror-pain synesthesia. Perspective taking involves the ability to understand and consider another person's thoughts, feelings, and point of view, while fantasy, on the other hand, involves the imaginative projection of oneself into another's situation or experience. However no cognitive dimension of empathy has been found to correlate with mirror-pain synesthesia experiences^7,15,18,19^ apart from empathic concern^16^. The role of trait empathy measurement adds another layer of complexity to this discussion. Trait empathy refers to stable individual differences in empathic tendencies, which can be measured using self-report questionnaires and are not designed to assess empathy while it changes in different contexts^47^. Future studies could additionally incorporate experimental manipulations to implicitly assess motivations for helping, whether driven by concern for the suffering other or by the desire to alleviate one's own distress elicited by witnessing the other's pain.

Neurally, in addition to finding that brain activity in regions associated with the affective components of pain were associated with donation (including the insula and cingulate for the Face and, at reduced threshold, for the Hand stimuli, in line with previous studies^3,13,26,27^), our results showed that donations were also associated with activity in somatosensory brain regions (SII and, when using a multivariate approach that has higher sensitivity than univariate analyses, we can additionally detect contribution of areas with small to medium effect sizes, SI). Additionally, TPJ and pSTS were found to have activity associated with the donation made in the Face condition, which dovetails with the notion that these areas play important roles in attention and theory of mind^48^ and dynamic facial expression encoding^49^. Moreover, as expected, we did find that donations were more tightly associated with activity in the somatosensory cortices (SII) for the self-report synesthetes. This latter finding is in line with previous reports that associate mirror synesthesia with increased somatosensory activation^6,38,39^, but extend this finding to prosociality.

In addition, using the neurological pain signature^40^, developed to quantify the recruitment of neural activity typical of feeling somatic pain on one’s own body, we could confirm that trials with higher donations (as captured by the HandDonation or FaceDonation parametric modulator) were associated with higher recruitment of this somatic pain pattern. Surprisingly, this was true for the Hand and Face stimuli, without significant differences between them, and was not more strongly the case for self-report synesthetes. This lack of specificity may be due to the holistic nature of the pattern that includes, but is not specific to, somatosensory brain regions^40^. That patterns trained to capture the recruitment of pain observation and guilt also overlap with those for costly helping, and do so in ways that do not depend on self-reported synesthesia, is perhaps less surprising given that several voxelwise studies have associated individual brain regions involved in affective empathy and guilt with prosociality^3,13,26,27,50^ and these more affective regions would be less expected to be associated with self-reported mirror pain synesthesia.

Together, our neuroscientific findings therefore support the notion that the degree to which observers recruit their own pain circuitry, including affective but particularly somatosensory pain components, is indeed associated with their willingness to sacrifice their own money to help others in pain. Our results also support the specific question of how much somatically feeling the pain of others onto our own body contributes to this willingness to help. Self-declared mirror-pain synesthetes, and to a lesser extend responders in the VPQ, do donate more money to alleviate the pain of others in our sample, and have a steeper donation slope (i.e. increase their donations more steeply as the pain of the victim increases). In addition, fMRI analysis revealed that activity in somatosensory cortices is associated with donation, as is the recruitment of patterns associated with somatic pain and activity in SII was more tightly associated with donation for these synesthetes when observing a hand being slapped. The latter suggests that if participants do somatically mirror the pain of others onto their own body, this leads to an increased motivation that is also more dependent on activity in their secondary somatosensory cortices.

Our study also has certain limitations that could inspire future studies. Firstly, we used self-reports of mirror pain experiences outside of the lab as our way to identify who is a mirror-pain synesthete. Philosophically, it is such subjective nociception in real life that is thought to motivate helping. However, future studies may wish to probe how much mirror pain participants feel on every trial within the experiment, and examine if variance in mirroring across trials in which similar levels of pain are observed can account for unique variance in helping. That the VPQ for instance does not lead to the exact same classification into mirror pain-synesthetes and controls (see Table 1) – although it’s classification also leads to significant differences in donation – reinforces the opportunity for more fine grained analysis and classifications of the subjective experience of mirror pain^10^ and its association with helping. Second, our neuroimaging findings only show significant correlations between brain activity (or multivariate patterns thereof) in somatosensory regions and donations, and cannot prove that such associations are causal in nature. In the past, we have shown that altering brain activity in SI non-invasively using TMS in participants can alter helping, specifically by altering how tightly participants tailor their helping to the needs of the target individual^14^. Using similar methodologies in participants reporting mirror pain synesthesia and measuring the effect on subjective feelings and helping will be key to a tighter understanding of the contribution of somatosensory cortices, the difference between SI and SII’s contribution, and the motivation to help. Finally, we only tested female participants in our paradigm. This was a decision we made based on extensive literature showing that synesthesia is by far more common in women^51–54^ even though there is some evidence that the difference is partly biased due to the fact the female participants are reacting more to experiment calls^55^. We were also aware that Gallo et al.^14^ using the same paradigm did not find any differences in donation between females and males. Future studies may however attempt to recruit more male mirror-touch synesthetes to explore if there might be sex-differences in the circuitry motivating helping, and in the contribution of somatosensory regions in particular. That preclinical studies have suggested substantial differences in the biological basis of nociception across male and female rodents is an intriguing reminder for the need of being mindful of the potential for sex-differences in pain-related phenomena^56^.

## Conclusion

To elucidate the impact of somatic experiences on costly helping, we tested individuals who self-reported experiencing the pain of others alongside those who did not, using a costly helping paradigm. During fMRI scanning, participants observed a confederate receiving painful stimuli, whose intensity they could reduce by donating money. Pain intensity was conveyed either through the facial expressions of the confederate or the movements of the pain-receiving hand. Our findings revealed heightened prosocial behavior and enhanced association of decision-making with somatosensory brain activity among self-reported mirror-pain synesthetes when the pain intensity was conveyed by the pained hand. These findings highlight the role of somatic experiences in guiding responses to others' distress in contexts requiring costly helping acts.

### Supplementary Information

Supplementary Information.

## Data Availability

Data are made available on OSF at https://osf.io/d3vyq/.
