# Supplementary material for: Neural mechanisms of costly helping in the general population and mirror-pain synesthetes
Source: Sci Rep. 2024 May 21;14:11617. doi: 10.1038/s41598-024-62422-3 (PMC11109206; doi:10.1038/s41598-024-62422-3)
Supplement: Supplementary file 1 — Supplementary Information. [file 41598_2024_62422_MOESM1_ESM.docx]

# **SUPPLEMENTARY INFORMATION S1**

## **Vicarious Pain Questionnaire**

## *Method*

The VPQ (Grice-Jackson et al., 2017) consists of 16 video clips of 10 seconds each depicting painful situations (e.g. injections and sporting injuries). After watching each video participants were asked if they felt any pain on their own body. In case they gave a positive response they were then asked additional questions: to rate the intensity (using a scale from 1 to 10), to report the location of the pain as it was felt (localized in same location as observed, localized in another location, a non localized general sensation), to choose any word from a list of pain adjectives that matched their vicarious pain experience. We followed the same two-step cluster analysis approach as in Grice-Jackson et al., 2017 which resulted in three different groups: affective generalized group A/G , sensory localized group S/L and non-responders. The tool was administered via LimeSurvey platform ([www.limesurvey.org](https://www.limesurvey.org/)). Three participants did not fill it in (one that had reported mirror pain synesthesia experiences and two that had not).

## *Results*

Out of the 31 participants that were included in our behavioral analyses, three participants did not complete the VPQ. Following the classification method of Grice-Jackson et al. (2017), of the 28 participants that did complete the VPQ, the distribution differed based on whether participants report mirror touch synesthesia or not (𝛘^2^(df=2)=7.032, *p*=0.03, BF_10_=5.283), with the likelihood to be classified as sensory/localizer 8 times higher in participants that reported mirror touch synesthesia than in those that do not (see Table S1). However, not all participants that self-report mirror touch synesthesia do qualify as responders.

**Table S1: VPQ classification as a function of self-report.** Each cell contains the number and proportion of participants falling into a specific VPQ classification (column) as a function of whether they do (top) or do not (bottom) self-report mirror pain synesthesia experiences in everyday life. A Chi-Square test on the contingency table confirms a significant difference in VPQ distribution based on self-report status, with self-reported mirror pain synesthetes having a higher proportion of sensory localizers and a lower proportion of non-responders (𝛘^2^(df=2)=7.032, *p*=0.03, BF_10_=5.283).

|  | **Sensory Localizers** | **Affective Generalizers** | **Non Responders** | **Missing VPQ** | **Total** |
| --- | --- | --- | --- | --- | --- |
| **Self-Report of Mirror Pain Synesthesia** | 6/12=50% | 1/12=8% | 5/12=42% | 1 | 13 |
| **No Self-Report of Mirror Pain Synesthesia** | 1/16=6% | 2/16=12% | 13/16=81% | 2 | 18 |

**Table S2: Correlations between IRI, MAS and the average donation that participants made.** The table summarizes the correlations for the average donation that participants made for the Face and Hand conditions together and the subscales of the IRI [(Davis and Association, 1980)](https://www.zotero.org/google-docs/?0url9i) (Fantasizing, Perspective Taking, Empathic Concern and Personal Distress) and MAS [(Yamauchi and Templer, 1982)](https://www.zotero.org/google-docs/?J9mxyv) (Power-Prestige, Retention-time, Distrust and Anxiety). None of these correlations were significant (all p>0.05) and all BFs<0.424 suggesting evidence for absence of an effect.

|  | **Scale** | **Pearson’s r** | **p** | **BF_10_** |
| --- | --- | --- | --- | --- |
| **IRI**  **Donation** | Fantasizing | 0.014 | 0.940 | 0.224 |
|  | Perspective Taking | 0.093 | 0.619 | 0.251 |
|  | Empathic Concern | 0.214 | 0.247 | 0.424 |
|  | Personal Distress | -0.045 | 0.810 | 0.230 |
| **MAS**  **Donation** | Power-Prestige | 0.001 | 0.995 | 0.223 |
|  | Retention-time | 0.145 | 0.436 | 0.299 |
|  | Distrust | -0.174 | 0.694 | 0.240 |
|  | Anxiety | -0.171 | 0.358 | 0.334 |

# **SUPPLEMENTARY INFORMATION S2**

## **Univariate GLM analyses**

When looking at the main effect of video1, i.e. voxels where the BOLD signal is increased while viewing the first video, independently of donation, and irrespectively of whether the pain was conveyed by the facial expression or the hand movement, we observed a network resembling the pain observation network often reported in the literature, including the ACC, MCC, SII and Insula (**Supplementary Fig. S1 and Supplementary Table S3**), suggesting that witnessing a painful stimulation delivered to the confederate triggered expected neural response. Comparing the main effect of Face and Hand during the the first video revealed significant differences across these two types of stimuli: the IFG and IPL showed higher BOLD signal for Face than Hand stimuli and SII, insula and the calcarine gyrus showed higher BOLD signal for the Hand than Face (**Supplementary Fig. S2 and Supplementary Table S4**). Comparing self-declared synesthetes and controls for the main effect of Face or Hand (i.e. independently of donation) did not yield significant differences.


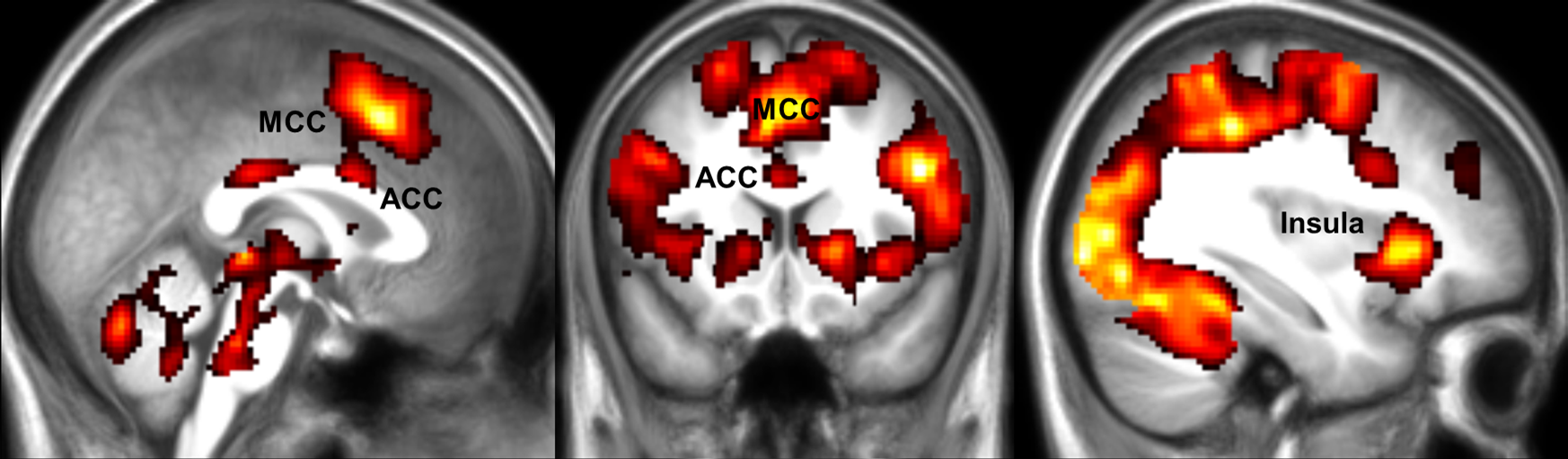


**Figure S1. Main effect of video 1.** Results of the main effects of Face and Hand conditions together, indicating voxels where BOLD signals during the pain observation are increased. Results are FWE cluster-corrected at p<0.05 (p<0.001, k=FWEc=389 voxels, cFWE 3.34<t<13).

**Table S3: Results of the voxelwise analysis. Brain activations for the main effect of video 1.** Regions were labeled using SPM Anatomy Toolbox. From left to right: the cluster size in number of voxels, the number of voxels falling in a cyto-architectonic area, the percentage of the cluster that falls in the cyto-architectonic area, the hemisphere (L=left; R=right), the name of the cyto-architectonic area when available or the anatomical description, the percentage of the area that is activated by the cluster, the t values of the peaks associated with the cluster followed by their MNI coordinates in mm.

| **Cluster size** | **# Voxels in cyto** | **% Cluster** | **Hem** | **Cyto or anatomical description** | **% Area** | **Peak Information** | | | |
| --- | --- | --- | --- | --- | --- | --- | --- | --- | --- |
|  |  |  |  |  |  | **T** | **x** | **y** | **z** |
| **Main effect of video 1 punc<0.001 k=FWEc=389** | | | | | | | | | |
| 49672 | 937 | 1.9 | L | Lobule VI (Hem) | 50 |  |  |  |  |
|  | 888.6 | 1.8 | R | Lobule VI (Hem) | 49.3 |  |  |  |  |
|  | 577.6 | 1.2 | L | Area 7A (SPL) | 46.1 |  |  |  |  |
|  | 560.4 | 1.1 | R | Area hOc1 [V1] | 27.1 |  |  |  |  |
|  | 557.5 | 1.1 | R | Area 2 | 85.8 |  |  |  |  |
|  | 530.3 | 1.1 | L | Area hOc4la  Inferior Occipital Gyrus | 62 | 14.93 | -46 | -72 | 2 |
|  | 512.5 | 1 | L | Area hOc4lp  Middle Occipital Gyrus | 59.9 | 14.83 | -40 | -86 | -2 |
|  | 497.6 | 1 | L | Area PFt (IPL) | 85.4 |  |  |  |  |
|  | 493.6 | 1 | R | Area hOc4la  Inferior Occipital Gyrus | 55.7 | 15.24 | 42 | -72 | -10 |
|  | 484.5 | 1 | L | Lobule VIIa crusI (Hem) | 15.9 |  |  |  |  |
|  | 452.6 | 0.9 | L | Area 44 | 51.7 |  |  |  |  |
|  | 448.3 | 0.9 | L | Area 2 | 85.1 |  |  |  |  |
|  | 446 | 0.9 | L | Area hOc1 [V1] | 22 |  |  |  |  |
|  | 443.1 | 0.9 | R | Thal: Prefrontal | 79.2 |  |  |  |  |
|  | 414.3 | 0.8 | R | Area FG4 | 84.6 |  |  |  |  |
|  | 409 | 0.8 | R | Area 44 | 68.2 |  |  |  |  |
|  | 390.5 | 0.8 | R | Area 1 | 55.6 |  |  |  |  |
|  | 390.5 | 0.8 | R | Area hIP3 (IPS) | 85.6 |  |  |  |  |
|  | 384.8 | 0.8 | R | Area hOc4v [V4(v)] | 61.9 |  |  |  |  |
|  | 378 | 0.8 | R | Lobule VIIa crusI (Hem) | 11.6 |  |  |  |  |
|  | 375.5 | 0.8 | L | Area hOc4v [V4(v)] | 51.7 |  |  |  |  |
|  | 363.5 | 0.7 | L | Area hIP3 (IPS) | 79.4 |  |  |  |  |
|  | 354 | 0.7 | L | Area FG4 | 59.9 |  |  |  |  |
|  | 353.4 | 0.7 | R | Area hOc3v [V3v] Inferior Occipital Gyrus | 41.5 | 16.28 | -20 | -92 | -6 |
|  | 330.1 | 0.7 | L | Thal: Prefrontal | 52.3 |  |  |  |  |
|  | 328.1 | 0.7 | R | Area PFt (IPL) | 78.7 |  |  |  |  |
|  | 319.6 | 0.6 | R | Area 3b | 50.8 |  |  |  |  |
|  | 308.1 | 0.6 | L | Area FG3 | 37.3 |  |  |  |  |
|  | 284.9 | 0.6 | R | Area PF (IPL) | 42.2 |  |  |  |  |
|  | 281.9 | 0.6 | R | Area hOc4lp | 50.4 | 14.56 | 38 | -86 | 4 |
|  | 280.5 | 0.6 | R | Area 7A (SPL) | 36 |  |  |  |  |
|  | 269.1 | 0.5 | R | Area 45 | 26.1 |  |  |  |  |
|  | 268.3 | 0.5 | R | Thal: Temporal | 49.1 |  |  |  |  |
|  | 254 | 0.5 | L | Area FG1  Inferior Occipital Gyrus | 99.6 | 14.92 | -36 | -74 | -8 |
|  | 251.3 | 0.5 | R | Area 7PC (SPL) | 55.3 |  |  |  |  |
|  | 247.1 | 0.5 | R | Area FG1 | 99.4 |  |  |  |  |
|  | 245.5 | 0.5 | L | Area hOc3v [V3v] | 26.5 |  |  |  |  |
|  | 229.1 | 0.5 | L | Area FG2 | 44.9 | 14.03 | -44 | -66 | -14 |
|  | 226.8 | 0.5 | L | Area 3b | 40.2 |  |  |  |  |
|  | 214.9 | 0.4 | R | Area FG3 | 32.8 |  |  |  |  |
|  | 208.4 | 0.4 | R | Thal: Parietal | 62.6 |  |  |  |  |
|  | 207.8 | 0.4 | L | Area hIP1 (IPS) | 57.1 |  |  |  |  |
|  | 204 | 0.4 | L | Thal: Temporal | 38.4 |  |  |  |  |
|  | 201.4 | 0.4 | R | Area FG2 | 61.9 |  |  |  |  |
|  | 197.5 | 0.4 | R | Area hIP2 (IPS) | 93.7 |  |  |  |  |
|  | 180.9 | 0.4 | R | Area 4p | 58.2 |  |  |  |  |
|  | 160.9 | 0.3 | L | Area 5L (SPL) | 23.2 |  |  |  |  |
|  | 151 | 0.3 | R | Area hIP1 (IPS) | 52.2 |  |  |  |  |
|  | 148 | 0.3 | L | Area hIP2 (IPS) | 65.9 |  |  |  |  |
|  | 140.5 | 0.3 | R | Area PFm (IPL) | 19.9 |  |  |  |  |
|  | 133 | 0.3 | R | Area hOc2 [V2] | 13 |  |  |  |  |
|  | 131.9 | 0.3 | L | Area 7PC (SPL) | 77.3 |  |  |  |  |
|  | 119.5 | 0.2 | L | Lobule VIIIa (Verm) | 80.7 |  |  |  |  |
|  | 118.5 | 0.2 | L | Thal: Parietal | 37.2 |  |  |  |  |
|  | 114.8 | 0.2 | R | Area PFcm (IPL) | 35.2 |  |  |  |  |
|  | 112.1 | 0.2 | R | Area 7P (SPL) | 23.8 |  |  |  |  |
|  | 107.6 | 0.2 | L | Area 1 | 18.9 |  |  |  |  |
|  | 105.9 | 0.2 | L | Lobule V (Hem) | 14.5 |  |  |  |  |
|  | 105.4 | 0.2 | L | Lobule VI (Verm) | 50.3 |  |  |  |  |
|  | 100.4 | 0.2 | L | Area hOc3d [V3d] | 10.1 |  |  |  |  |
|  | 99.4 | 0.2 | R | Area hOc3d [V3d] | 18.1 |  |  |  |  |
|  | 96.8 | 0.2 | L | Area 7P (SPL) | 27.8 |  |  |  |  |
|  | 95.8 | 0.2 | L | Area PFop (IPL) | 43.1 |  |  |  |  |
|  | 94.4 | 0.2 | L | Area PF (IPL) | 18.1 |  |  |  |  |
|  | 94.3 | 0.2 | R | Lobule VI (Verm) | 40.6 |  |  |  |  |
|  | 90 | 0.2 | L | Lobule VIIb (Hem) | 13.3 |  |  |  |  |
|  | 89.4 | 0.2 | R | Area 5L (SPL) | 12.2 |  |  |  |  |
|  | 82.4 | 0.2 | R | Lobule VIIIa (Verm) | 39.3 |  |  |  |  |
|  | 82 | 0.2 | L | Area hOc5 [V5/MT] | 102 |  |  |  |  |
|  | 78.5 | 0.2 | L | Lobule IX (Hem) | 12.6 |  |  |  |  |
|  | 76.5 | 0.2 | R | Area PGa (IPL) | 10.3 |  |  |  |  |
|  | 73.6 | 0.1 | R | Area 3a | 36.6 |  |  |  |  |
|  | 73 | 0.1 | R | Thal: Premotor | 54.9 |  |  |  |  |
|  | 72.1 | 0.1 | L | Lobule VIIa crusII (Hem) | 4.4 |  |  |  |  |
|  | 66.9 | 0.1 | L | Lobule IX (Verm) | 74.6 |  |  |  |  |
|  | 66.9 | 0.1 | R | Lobule VIIb (Hem) | 10.2 |  |  |  |  |
|  | 65.6 | 0.1 | L | Area hOc4d [V3A] | 11.5 |  |  |  |  |
|  | 62.1 | 0.1 | R | Area hOc4d [V3A] | 14.8 |  |  |  |  |
|  | 58.3 | 0.1 | R | Area hOc5 [V5/MT] | 100 |  |  |  |  |
|  | 55.1 | 0.1 | L | Thal: Visual | 61.5 |  |  |  |  |
|  | 53.8 | 0.1 | R | Area 4a | 4.9 |  |  |  |  |
|  | 48.3 | 0.1 | L | Area hOc2 [V2] | 5.1 |  |  |  |  |
|  | 45.9 | 0.1 | L | Area OP1 [SII] | 12.3 |  |  |  |  |
|  | 44.6 | 0.1 | R | Lobule IX (Verm) | 42.6 |  |  |  |  |
|  | 42.5 | 0.1 | L | Area 3a | 14.8 |  |  |  |  |
|  | 41.6 | 0.1 | R | Area PGp (IPL) | 4.2 |  |  |  |  |
|  | 41.5 | 0.1 | L | Lobule VIIIa (Hem) | 5.5 |  |  |  |  |
|  | 38.4 | 0.1 | R | Thal: Visual | 92.2 |  |  |  |  |
|  | 36.5 | 0.1 | L | Area PGp (IPL) | 4.4 |  |  |  |  |
|  | 36 | 0.1 | L | Lobule VIIb (Verm) | 117.6 |  |  |  |  |
|  | 34.8 | 0.1 | R | Lobule IX (Hem) | 4.9 |  |  |  |  |
|  | 34.1 | 0.1 | L | Area OP4 [PV] | 9.4 |  |  |  |  |
|  | 32.8 | 0.1 | R | Lobule VIIb (Verm) | 100 |  |  |  |  |
|  | 32.4 | 0.1 | R | Area PFop (IPL) | 14.2 |  |  |  |  |
|  | 31.6 | 0.1 | L | Area 45 | 4.5 |  |  |  |  |
|  | 30.9 | 0.1 | R | Thal: Somatosensory | 38 |  |  |  |  |
|  | 30.4 | 0.1 | L | Lobule I IV (Hem) | 6.3 |  |  |  |  |
|  | 26.3 | 0.1 | L | Area PFcm (IPL) | 8.1 |  |  |  |  |
|  | 24.5 | 0 | R | Subiculum | 6.5 |  |  |  |  |
|  | 23.1 | 0 | R | Lobule VIIIa (Hem) | 3.2 |  |  |  |  |
|  | 23 | 0 | L | Area 33 | 10.8 |  |  |  |  |
|  | 20.4 | 0 | L | Lobule VIIIb (Verm) | 33 |  |  |  |  |
|  | 20.3 | 0 | R | Lobule V (Hem) | 2.5 |  |  |  |  |
|  | 19.9 | 0 | R | Lobule I IV (Hem) | 4 |  |  |  |  |
|  | 19 | 0 | R | Lobule VIIa crusII (Hem) | 1.3 |  |  |  |  |
|  | 18.3 | 0 | R | Area 33 | 8.4 |  |  |  |  |
|  | 17.1 | 0 | L | Lobule VIIIb (Hem) | 2.8 |  |  |  |  |
|  | 16.1 | 0 | R | Lobule VIIa crusII (Verm) | 28.4 |  |  |  |  |
|  | 10.5 | 0 | R | Lobule VIIIb (Hem) | 1.5 |  |  |  |  |
|  | 10.5 | 0 | R | Area 5M (SPL) | 3.6 |  |  |  |  |
|  | 9.1 | 0 | R | DG (Hippocampus) | 7.1 |  |  |  |  |
|  | 8.5 | 0 | L | Area PFm (IPL) | 1.5 |  |  |  |  |
|  | 8.1 | 0 | L | Lobule VIIa crusII (Verm) | 18.2 |  |  |  |  |
|  | 7.8 | 0 | L | Area 4p | 2.4 |  |  |  |  |
|  | 7.6 | 0 | R | Area 7M (SPL) | 7.5 |  |  |  |  |
|  | 6.9 | 0 | R | Thal: Motor | 15.5 |  |  |  |  |
|  | 5.8 | 0 | R | BF (Ch 4) | 13.8 |  |  |  |  |
|  |  |  | R | IFG (p. Opercularis) |  | 15.60 | 48 | 14 | 28 |
|  |  |  | R | Posterior-Medial Frontal |  | 14.84 | 4 | 16 | 46 |
| 417 | 5.6 | 1.3 | L | Area 45 | 0.8 |  |  |  |  |
|  |  |  | L | Middle Frontal Gyrus |  | 6.71 | -44 | 38 | 26 |
|  |  |  | L | IFG (p. Triangularis) |  | 5.90 | -40 | 34 | 16 |
|  |  |  | L | Middle Frontal Gyrus |  | 4.08 | -42 | 48 | 12 |
| 389 | 1.4 | 0.4 | R | Area 33 | 0.6 |  |  |  |  |

Face - Hand Hand - Face


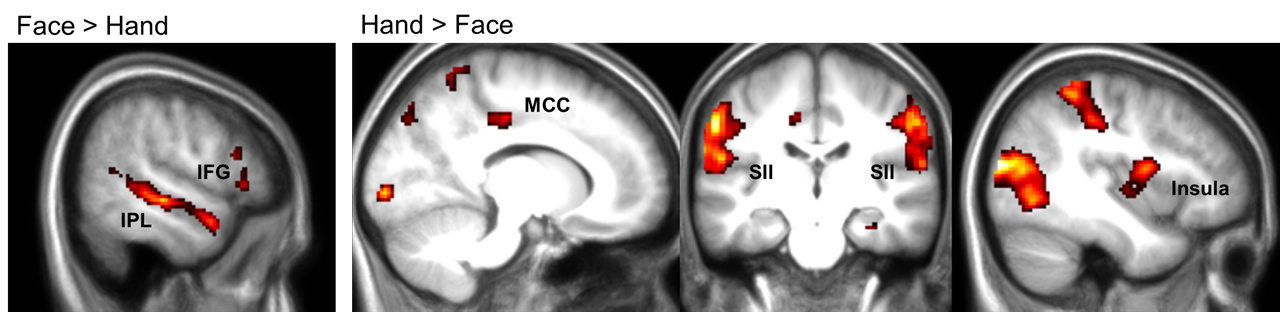
**Figure S2. Effect of stimulus type (Face vs Hand).** Comparison between the face and hand conditions for the first video pain observation. Results are FWE cluster-corrected at p<0.05 (p_unc_<0.001, k=FWEc=145 voxels for the face and k=FWEc=125 voxels for the hand). For this analysis we constructed a different GLM, same to the one described at the *GLM analysis* methods section, without any parametric modulators.

**Table S4: Results of the voxelwise analysis. Brain activations for the effect of stimulus type (Face vs Hand).** Regions were labeled using SPM Anatomy Toolbox. From left to right: the cluster size in number of voxels, the number of voxels falling in a cyto-architectonic area, the percentage of the cluster that falls in the cyto-architectonic area, the hemisphere (L=left; R=right), the name of the cyto-architectonic area when available or the anatomical description, the percentage of the area that is activated by the cluster, the t values of the peaks associated with the cluster followed by their MNI coordinates in mm.

| **Cluster size** | **# Voxels in cyto** | **% Cluster** | **Hem** | **Cyto or anatomical description** | **% Area** | **Peak Information** | | | |
| --- | --- | --- | --- | --- | --- | --- | --- | --- | --- |
|  |  |  |  |  |  | **T** | **X** | **Y** | **Z** |
| **Effect of stimulus type (Face vs Hand) video 1 punc<0.001 k=FWEc=145** | | | | | | | | | |
| 1098 | 63.9 | 5.8 | R | Area TE 3 Middle Temporal Gyrus | 5.8 | 5.32 | 58 | 0 | -16 |
|  | 25.9 | 2.4 | R | Area PGa (IPL) Superior Temporal Gyrus | 3.3 | 3.88 | 62 | -52 | 18 |
|  | 6.1 | 0.6 | R | Area Id1 | 3.5 |  |  |  |  |
| 242 | 9.5 | 3.9 | L | Area TE 3 | 1.1 |  |  |  |  |
|  |  |  | L | Middle Temporal Gyrus |  | 4.63 | -52 | -20 | -4 |
| 240 | 110.6 | 46.1 | R | Area 45 IFG (p. Triangularis) | 10.7 | 4.63 | 56 | 22 | 2 |
|  | 6.1 | 2.6 | R | Area 44 | 1 |  |  |  |  |
|  |  |  | R | IFG (p. Orbitalis) |  | 4.13 | 46 | 28 | -4 |
| 145 |  |  | R | Superior Medial Gyrus |  | 5.26 | 8 | 28 | 56 |
|  |  |  | R | Posterior-Medial Frontal |  | 4.87 | 10 | 10 | 71 |
| **Effect of stimulus type (Hand vs Face) video 1 punc<0.001 k=FWEc=125** | | | | | | | | | |
| 8735 | 581 | 6.7 | R | Area 2 | 89.4 | 7.88 | 32 | -38 | 54 |
|  | 342.3 | 3.9 | R | Area 3b | 54.4 |  |  |  |  |
|  | 299.1 | 3.4 | R | Area hOc1 [V1] Calcarine Gyrus | 14.5 | 7.26 | 20 | -94 | 0 |
|  | 296.1 | 3.4 | R | Area hOc3v [V3v] | 34.7 |  |  |  |  |
|  | 282.3 | 3.2 | R | Area PFt (IPL) | 67.7 |  |  |  |  |
|  | 282 | 3.2 | R | Area 1 Postcentral Gyrus | 40.1 | 7.48 | 60 | -18 | 36 |
|  | 265.1 | 3 | R | Area FG4 Fusiform Gyrus | 54.2 | 7.82 | 30 | -42 | -12 |
|  | 247.8 | 2.8 | R | Area hOc4la | 27.9 |  |  |  |  |
|  | 237.1 | 2.7 | R | Area hOc4v [V4(v)] | 38.1 |  |  |  |  |
|  | 226.9 | 2.6 | R | Area 5L (SPL) | 30.9 |  |  |  |  |
|  | 222.6 | 2.5 | R | Area 7PC (SPL) | 49 |  |  |  |  |
|  | 183.3 | 2.1 | R | Area OP1 [SII] | 46.9 |  |  |  |  |
|  | 181.9 | 2.1 | R | Area hOc4lp Middle Occipital Gyrus | 32.5 | 7.44 | 38 | -88 | 6 |
|  | 160.6 | 1.8 | R | Area hOc4d [V3A] | 38.2 |  |  |  |  |
|  | 147.1 | 1.7 | R | Area PFop (IPL) | 64.3 |  |  |  |  |
|  | 119.1 | 1.4 | R | Area 7A (SPL) | 15.3 |  |  |  |  |
|  | 113 | 1.3 | R | Area PFcm (IPL) | 34.7 |  |  |  |  |
|  | 104.1 | 1.2 | R | Lobule VI (Hem) | 5.8 |  |  |  |  |
|  | 100.6 | 1.2 | R | Area hIP3 (IPS) | 22.1 |  |  |  |  |
|  | 90.5 | 1 | R | Area FG1 | 36.4 |  |  |  |  |
|  | 88.6 | 1 | R | Area 4a | 8.1 |  |  |  |  |
|  | 82.9 | 0.9 | R | Area 5Ci (SPL) | 41.9 |  |  |  |  |
|  | 81.5 | 0.9 | R | Area 7P (SPL) | 17.3 |  |  |  |  |
|  | 80.5 | 0.9 | R | Area 4p | 25.9 |  |  |  |  |
|  | 65.5 | 0.7 | R | Area PF (IPL) | 9.7 |  |  |  |  |
|  | 65.3 | 0.7 | R | Area hOc2 [V2] | 6.4 |  |  |  |  |
|  | 51.9 | 0.6 | R | Area hOc5 [V5/MT] | 89.1 |  |  |  |  |
|  | 44 | 0.5 | R | Area 3a | 21.8 |  |  |  |  |
|  | 38.1 | 0.4 | R | Area 5M (SPL) | 13 |  |  |  |  |
|  | 35.9 | 0.4 | R | Area PGp (IPL) | 3.6 |  |  |  |  |
|  | 32.3 | 0.4 | R | Area hOc3d [V3d] | 5.9 |  |  |  |  |
|  | 29.5 | 0.3 | R | CA1 (Hippocampus) | 10.2 |  |  |  |  |
|  | 22.6 | 0.3 | R | Area FG2 | 7 |  |  |  |  |
|  | 14 | 0.2 | R | Area hIP2 (IPS) | 6.6 |  |  |  |  |
|  | 13.5 | 0.2 | R | Lobule V (Hem) | 1.7 |  |  |  |  |
|  | 11.8 | 0.1 | R | Subiculum | 3.1 |  |  |  |  |
|  | 6.4 | 0.1 | R | Area TE 1.1 | 3.2 |  |  |  |  |
|  | 3.9 | 0 | R | Area hIP1 (IPS) | 1.3 |  |  |  |  |
|  | 3.5 | 0 | R | CA2 (Hippocampus) | 5.8 |  |  |  |  |
|  | 3.1 | 0 | R | DG (Hippocampus) | 2.4 |  |  |  |  |
|  | 1.6 | 0 | R | Area TE 3 | 0.2 |  |  |  |  |
|  | 1.4 | 0 | R | Area PFm (IPL) | 0.2 |  |  |  |  |
|  | 0.9 | 0 | R | Lobule VIIa crusI (Hem) | 0 |  |  |  |  |
|  |  |  | R | Inferior Temporal Gyrus |  | 7.36 | 46 | -62 | -4 |
| 7696 | 411.9 | 5.4 | L | Area 2 Postcentral Gyrus | 78.2 | 8.04 | -32 | -42 | 54 |
|  | 405.9 | 5.3 | L | Area PFt (IPL) SupraMarginal Gyrus | 69.6 | 7.92 | -58 | -24 | 38 |
|  | 394.1 | 5.1 | L | Area FG4 Fusiform Gyrus | 66.7 | 8.51 | -28 | -50 | -8 |
|  | 357.5 | 4.6 | L | Area 5L (SPL) | 51.5 |  |  |  |  |
|  | 342.5 | 4.5 | L | Area hOc4la Middle Occipital Gyru | 40.1 | 8.11 | -48 | -70 | -2 |
|  | 305.1 | 4 | L | Area 7A (SPL) | 24.4 |  |  |  |  |
|  | 298.6 | 3.9 | L | Area hOc4lp Middle Occipital Gyrus | 34.9 | 8.07 | -40 | -84 | 14 |
|  | 280 | 3.6 | L | Area OP1 [SII] | 75.2 |  |  |  |  |
|  | 226.9 | 2.9 | L | Area 3b Postcentral Gyrus | 40.2 | 7.45 | -58 | -14 | 30 |
|  | 186.4 | 2.4 | L | Area PFop (IPL) | 83.9 |  |  |  |  |
|  | 183.8 | 2.4 | L | Lobule VI (Hem) | 9.8 |  |  |  |  |
|  | 183.4 | 2.4 | L | Area PFcm (IPL) | 56.6 |  |  |  |  |
|  | 156.4 | 2 | L | Area FG1 Fusiform Gyrus | 61.3 | 7.68 | -30 | -58 | -16 |
|  | 129.6 | 1.7 | L | Area 7PC (SPL) | 76 |  |  |  |  |
|  | 128.8 | 1.7 | L | Area PF (IPL) | 24.6 |  |  |  |  |
|  | 115.4 | 1.5 | L | Area hIP3 (IPS) | 25.2 |  |  |  |  |
|  | 113.4 | 1.5 | L | Area hOc4v [V4(v)] | 15.6 |  |  |  |  |
|  | 105.8 | 1.4 | L | Area 1 | 18.6 |  |  |  |  |
|  | 89.6 | 1.2 | L | Area OP4 [PV] | 24.8 |  |  |  |  |
|  | 80.4 | 1 | L | Area hOc5 [V5/MT] | 100 |  |  |  |  |
|  | 80.1 | 1 | L | Area hOc4d [V3A] | 14 |  |  |  |  |
|  | 75.3 | 1 | L | Area PGp (IPL) | 9.1 |  |  |  |  |
|  | 46.3 | 0.6 | L | Area 3a | 16.1 |  |  |  |  |
|  | 28.6 | 0.4 | L | Area TE 3 | 3.2 |  |  |  |  |
|  | 28.3 | 0.4 | L | Area 4p | 8.7 |  |  |  |  |
|  | 26.5 | 0.3 | L | Area FG2 | 5.2 |  |  |  |  |
|  | 22.5 | 0.3 | L | Lobule V (Hem) | 3.1 |  |  |  |  |
|  | 14 | 0.2 | L | Area 4a | 1.5 |  |  |  |  |
|  | 14 | 0.2 | L | Area FG3 | 1.7 |  |  |  |  |
|  | 11.4 | 0.1 | L | Area TE 1.1 | 7.1 |  |  |  |  |
|  | 6.9 | 0.1 | L | Area 7P (SPL) | 2 |  |  |  |  |
|  | 5.6 | 0.1 | L | Area hIP1 (IPS) | 1.5 |  |  |  |  |
|  | 5.5 | 0.1 | L | CA1 (Hippocampus) | 2.5 |  |  |  |  |
|  | 5.4 | 0.1 | L | Area TE 1.0 | 4.3 |  |  |  |  |
|  | 5.1 | 0.1 | L | Area 5M (SPL) | 1.1 |  |  |  |  |
|  | 4.4 | 0.1 | L | Area OP3 [VS] | 3.1 |  |  |  |  |
|  | 3 | 0 | L | Subiculum | 0.8 |  |  |  |  |
|  | 2.1 | 0 | L | Area hIP2 (IPS) | 0.9 |  |  |  |  |
|  | 1.1 | 0 | L | Area hOc3d [V3d] | 0.1 |  |  |  |  |
| 322 | 3.1 | 1 | L | Area TE 1.2 Superior Temporal Gyrus | 2.2 | 4.54 | -48 | 0 | -2 |
|  | 2.6 | 0.8 | L | Area 44 | 0.3 |  |  |  |  |
|  | 1.4 | 0.4 | L | Area OP3 [VS] | 1 |  |  |  |  |
|  | 0.5 | 0.2 | L | Area Ig2 | 0.4 |  |  |  |  |
|  | 0.3 | 0.1 | L | Area OP4 [PV] | 0.1 |  |  |  |  |
|  | 0.1 | 0 | L | Area TE 3 | 0 |  |  |  |  |
|  |  |  | L | Insula Lobe |  | 6 | -40 | -2 | 12 |
|  |  |  | L | Rolandic Operculum |  | 5.92 | -38 | 2 | 14 |
|  |  |  | L | L IFG (p. Opercularis) |  | 4.65 | -52 | 6 | 6 |
| 153 | 84.4 | 55.1 | L | Area hOc1 [V1] Calcarine Gyrus | 4.2 | 6.92 | -14 | -94 | -2 |
|  | 18 | 11.8 | L | Area hOc4lp | 2.1 |  |  |  |  |
|  | 11 | 7.2 | L | Area hOc3v [V3v] | 1.2 |  |  |  |  |
|  | 10 | 6.5 | L | Area hOc3d [V3d] Middle Occipital Gyrus | 1 | 4.01 | -22 | -98 | 6 |
|  | 1.6 | 1.1 | L | Area hOc2 [V2] | 0.2 |  |  |  |  |
| 125 | 32 | 25.6 | L | Area 5Ci (SPL) | 27.2 |  |  |  |  |
|  | 0.9 | 0.7 | L | Area 5M (SPL) | 0.2 |  |  |  |  |
|  |  |  | L | MCC |  | 5.05 | -12 | -24 | 42 |
|  |  |  |  |  |  |  |  |  |  |


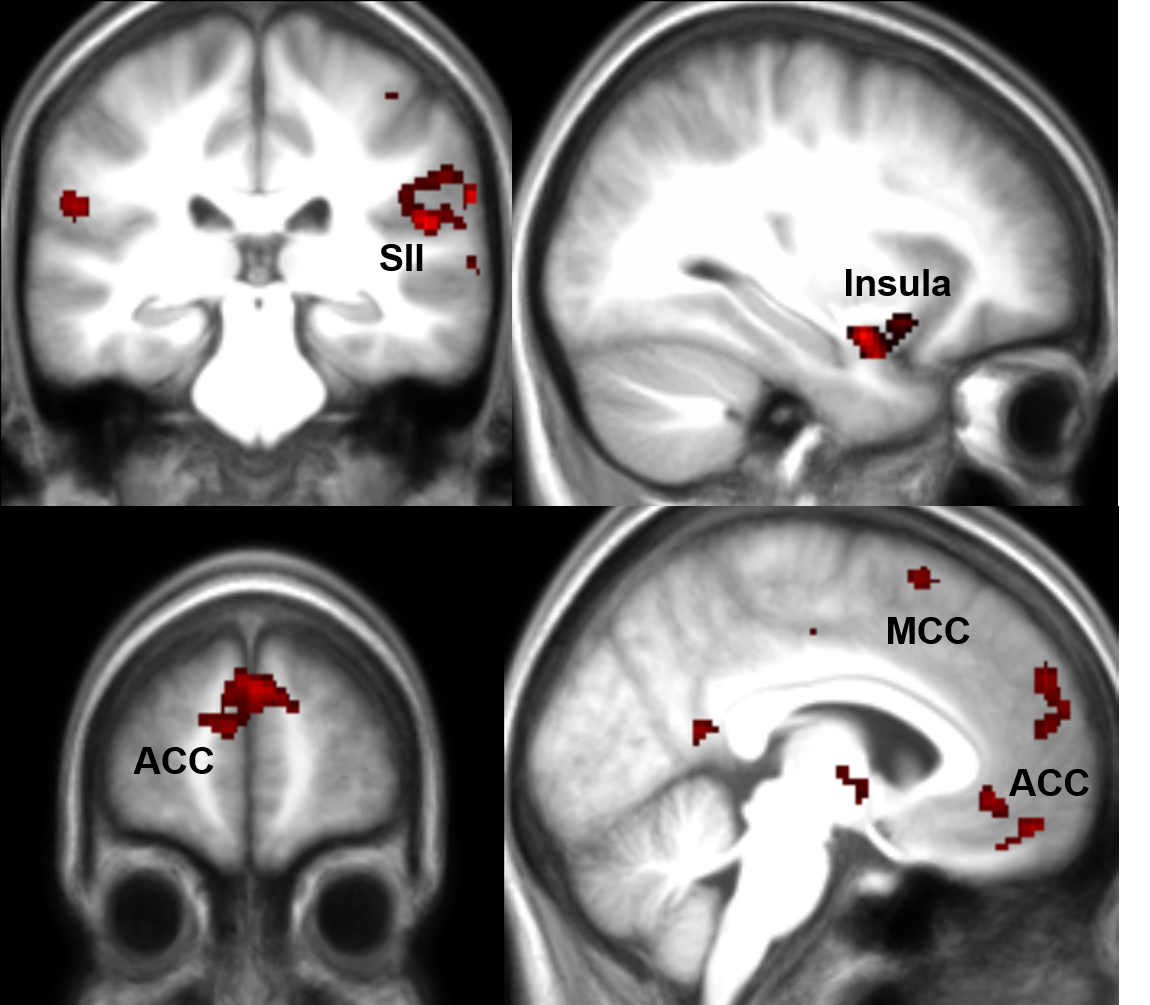


**Figure S3. HandDonation parametric modulator at reduced threshold.** Results of a linear regression on the parametric modulator for the first video and trial-by-trial donation in the Hand condition. This identifies voxels with signals that increase for higher donation. Results are shown at uncorrected p<0.01, 2.4<t<8.

**Table S5: Results of the voxelwise analysis. Brain activations for the HandDonation parametric modulator for all participants together at reduced threshold.** Regions were labeled using SPM Anatomy Toolbox. From left to right: the cluster size in number of voxels, the number of voxels falling in a cyto-architectonic area, the percentage of the cluster that falls in the cyto-architectonic area, the hemisphere (L=left; R=right), the name of the cyto-architectonic area when available or the anatomical description, the percentage of the area that is activated by the cluster, the t values of the peaks associated with the cluster followed by their MNI coordinates in mm.

| **Cluster size** | **# Voxels in cyto** | **% Cluster** | **Hem** | **Cyto or anatomical description** | **% Area** | **Peak Information** | | | | |
| --- | --- | --- | --- | --- | --- | --- | --- | --- | --- | --- |
|  |  |  |  |  |  | **T** | **x** | **y** | | **z** |
| **HandDonation at reduced threshold punc<0.01 k=15** | | | | | | | | | | |
| 712 | 53.8 | 7.5 | R | Area TE 3 Middle Temporal Gyrus | 5.2 | 4.72 | 58 | | 0 | -14 |
|  | 2.5 | 0.4 | R | Area Fo2 | 0.2 |  |  | |  |  |
|  | 1 | 0.1 | R | Area Id1 | 0.6 |  |  | |  |  |
|  |  |  | R | IFG (p. Orbitalis) |  | 3.62 | 32 | | 14 | -22 |
| 539 | 158.5 | 29.4 | R | Area PFcm (IPL) | 158.5 | 4.24 | 52 | | -30 | 16 |
|  | 143.6 | 26.6 | R | Area PF (IPL) | 143.6 | 4.97 | 60 | | -36 | 28 |
|  | 27.9 | 5.2 | R | Area OP1 [SII] | 27.9 |  |  | |  |  |
|  | 21 | 3.9 | R | Area PFm (IPL) | 21 |  |  | |  |  |
|  | 8.3 | 1.5 | R | Area TE 3 Superior Temporal Gyrus | 8.3 | 2.76 | 66 | | -32 | 4 |
|  | 3.9 | 0.7 | R | Area PFt (IPL) | 3.9 |  |  | |  |  |
|  | 1.3 | 0.2 | R | Area PGa (IPL) | 1.3 |  |  | |  |  |
|  | 0.8 | 0.1 | R | Area PFop (IPL) | 0.8 |  |  | |  |  |
| 331 | 14.1 | 4.3 | L | Area Id1 | 12 |  |  | |  |  |
|  | 4.6 | 1.4 | L | Area TE 1.2 | 3.3 | 3 | -52 | | -2 | -4 |
|  | 0.5 | 0.2 | L | Area TE 3 | 0.1 |  |  | |  |  |
| 236 |  |  | R | Posterior-Medial Frontal |  | 3.87 | 10 | | 6 | 66 |
|  |  |  | L | Posterior-Medial Frontal |  |  |  | |  |  |
| 233 | 13.8 | 5.9 | L | Area Fp2 | 1.9 |  |  | |  |  |
|  | 1.8 | 0.8 | R | Area Fp2 | 0.3 |  |  | |  |  |
|  | 0.1 | 0.1 | L | Area Fp1 | 0 |  |  | |  |  |
|  |  |  | R | Superior Medial Gyrus |  | 3.90 | 4 | | 58 | 26 |
|  |  |  | L | Superior Medial Gyrus |  | 3.37 | -6 | | 54 | 26 |
| 231 |  |  | L | IFG (p. Triangularis) |  |  | -40 | | 22 | 3 |
|  |  |  | L | IFG (p. Orbitalis) |  |  | -42 | | 20 | -7 |
| 218 | 42.5 | 19.5 | L | Amygdala (LB) | 17.5 |  |  | |  |  |
|  | 21.3 | 9.7 | L | Amygdala (CM) | 49.1 |  |  | |  |  |
|  | 8.9 | 4.1 | L | Amygdala (AStr) | 41.3 |  |  | |  |  |
|  | 2.3 | 1 | L | BF (Ch 4) | 4.5 |  |  | |  |  |
|  | 1.8 | 0.8 | L | CA1 (Hippocampus) | 0.8 |  |  | |  |  |
|  | 1.3 | 0.6 | L | HATA Region | 5.3 |  |  | |  |  |
|  |  |  | L | Insula Lobe |  | 3.16 | -30 | | 10 | -14 |
| 102 | 30 | 29.4 | R | Area 45 IFG (p. Triangularis) | 2.9 | 3.72 | 52 | | 26 | 0 |
|  |  |  | R | R IFG (p. Orbitalis) |  | 3.21 | 46 | | 28 | -4 |
| 88 | 34.9 | 39.6 | L | Area Fp2 Rectal Gyrus | 4.8 | 3.98 | -6 | | 50 | -16 |
|  | 24.9 | 28.3 | L | Area Fo1 Rectal Gyrus | 5.3 | 2.99 | -4 | | 42 | -22 |
|  | 4.1 | 4.7 | R | Area Fo1 | 0.8 |  |  | |  |  |
|  | 2.4 | 2.7 | R | Area Fp2 | 0.4 |  |  | |  |  |
| 83 | 39.4 | 47.4 | L | Area PFop (IPL) | 17.7 | 4.31 | -54 | | -28 | 22 |
|  | 16 | 19.3 | L | Area OP1 [SII] | 4.3 |  |  | |  |  |
|  | 12.5 | 15.1 | L | Area PFcm (IPL) | 3.9 |  |  | |  |  |
|  | 11.4 | 13.7 | L | Area PF (IPL) | 2.2 | 3.03 | -60 | | -34 | 24 |
| 82 | 47.4 | 57.8 | L | Area hOc4la | 5.5 | 3.62 | -48 | | -80 | 4 |
|  | 11.1 | 13.6 | L | Area hOc5 [V5/MT] | 13.8 |  |  | |  |  |
| 62 | 30 | 43.5 | L | Area s32 | 14.3 |  |  | |  |  |
|  | 8.8 | 12.7 | L | Area s24 | 5.2 |  |  | |  |  |
|  | 5 | 7.2 | R | Area s32 | 3.4 |  |  | |  |  |
|  | 4.9 | 7.1 | R | Area s24 ACC | 3.3 | 2.50 | 2 | | 32 | -4 |
|  | 2.6 | 3.8 | R | Area 33 ACC | 1.2 | 2.50 | 4 | | 32 | 0 |
|  |  |  | L | ACC |  | 3.42 | -2 | | 38 | -6 |
| 58 | 6.3 | 10.8 | R | Area 5Ci (SPL) | 3.2 |  |  | |  |  |
|  | 0.5 | 0.9 | R | Area 5M (SPL) | 0.2 |  |  | |  |  |
| 57 | 0.1 | 0.2 | R | Area 3b | 0 |  |  | |  |  |
| 46 | 7.8 | 16.8 | L | Thal: Temporal | 1.5 |  |  | |  |  |
|  | 6.5 | 14.1 | R | Thal: Temporal | 1.2 | 3.67 | 4 | | -6 | -2 |
|  | 3.8 | 8.2 | L | Thal: Prefrontal | 0.6 | 2.74 | -4 | | -8 | 0 |
|  | 2.6 | 5.7 | R | Thal: Prefrontal | 0.5 |  |  | |  |  |
| 42 | 10.4 | 24.7 | L | Area hOc4d [V3A] | 1.8 |  |  | |  |  |
| 39 | 29.6 | 76 | R | Area 3b | 4.7 | 3.42 | 36 | | -32 | 52 |
|  | 5.3 | 13.5 | R | Area 4p | 1.7 |  |  | |  |  |
|  | 2.1 | 5.4 | R | Area 1 | 0.3 |  |  | |  |  |
|  | 1.6 | 4.2 | R | Area 4a | 0.1 |  |  | |  |  |
|  | 0.3 | 0.6 | R | Area 2 | 0 |  |  | |  |  |
|  | 0.1 | 0.3 | R | Area 3a | 0.1 |  |  | |  |  |
| 39 | 17.9 | 45.8 | R | Amygdala (LB) | 8.4 |  |  | |  |  |
|  | 6.9 | 17.6 | R | Amygdala (SF) | 14.4 |  |  | |  |  |
|  | 2.3 | 5.8 | R | HATA Region | 10.3 |  |  | |  |  |
|  | 0.8 | 1.9 | R | CA1 (Hippocampus) | 0.3 |  |  | |  |  |
|  | 0.5 | 1.3 | R | Amygdala (CM) | 1.8 |  |  | |  |  |
| 38 | 37.3 | 98 | R | Area PGp (IPL) | 3.8 | 4.10 | 54 | | -68 | 18 |
|  | 0.8 | 2 | R | Area hOc4la | 0.1 |  |  | |  |  |
| 30 | 16.6 | 55.4 | R | Area hOc5 [V5/MT] | 28.5 |  |  | |  |  |
|  | 11.3 | 37.5 | R | Area hOc4la | 1.3 |  |  | |  |  |
| 24 | 5.6 | 23.4 | R | Area PGp (IPL) | 0.6 |  |  | |  |  |
|  | 0.3 | 1 | R | Area PGa (IPL) | 0 |  |  | |  |  |
| 20 | 17.3 | 86.3 | R | Area FG3 | 2.6 | 3.71 | 40 | | -48 | -18 |
|  | 1.8 | 8.8 | R | Area FG4 | 0.4 |  |  | |  |  |
| 19 |  |  | R | MCC |  | 2.91 | 10 | | -22 | 46 |
|  |  |  | L | MCC |  | 2.72 |  | |  |  |
| 19 |  |  | L | Precuneus |  | 3.24 | -4 | | -56 | 19 |
| 16 | 11 | 68.8 | R | Area OP1 [SII] | 2.8 |  |  | |  |  |
|  | 0.1 | 0.8 | R | Area OP3 [VS] | 0.1 |  |  | |  |  |
|  | 0.1 | 0.8 | R | Area PFop (IPL) | 0.1 |  |  | |  |  |

#

# **SUPPLEMENTARY INFORMATION S3**

## **Multivariate fMRI analysis**

*Methods*

For each participant, we performed a general linear model that estimated a separate parameter estimate for the activity during movie 1 for each level of donation (0-6) that participants made at least two times during the experiment. In case a level of donation occurred just once, it was added in a regressor of no interest and was not analyzed further. Using the anatomy toolbox, we then created an ROI containing all voxels attributed to SI according to the maximum probability maps (i.e. including bilateral BA3a, 3b, 1 and 2), and transformed this mask into the space of the parameter estimates using imagecalc. Using matlab, we then loaded for each subject the parameter estimate images for each level of donation, and only included voxels that fell within our SI mask. Next, we performed a weighted leave-one-subject-out cross-validated partial least square regression. For each of the 29 participants, we kept one subject out, and used the function plsregress in matlab to determine the linear combination of voxels that best predicts donation in the remaining participants. Because some parameter estimates derived from only two trials, and others from as many as 22 trials, we weighted the regression by replicating each parameter estimate image in the training and testing set by the number of trials that went into it. We then used this optimal linear combination to predict the donation of the left-out participant, and quantified the accuracy of the prediction as the correlation between predicted and actual donations. We used Kendall’s Tau as the measure of correlation because it is less susceptible to outliers as a parametric correlation. For the pls-regression, results are shown for using 10 components, based on the elbow method of explained variance including the entire dataset, but results are stable over a range of 8-20 components. We also performed a PCR by first performing a principle component analysis on all the voxel parameter estimates, and then using the first 10 components to perform leave one out regressions to predict donation. This also led to above chance estimates, but in the paper we only report the partial least square regression approach.

*Results*

To explore if SI (i.e. BA3a,3b,1,2) contains information about donation also for the hand trials, for which we failed to find significant evidence at the univariate level that survives correction, we performed a multivariate analysis. Specifically, we trained a weighted partial least-square regression using the data from all but one participant to estimate donation based on a linear combination of the parameter estimates in each voxel in SI, and then used this linear combination to predict the donations of the left-out participant (i.e. a leave one subject out cross-validation). We then quantified how accurately the regression predicted the donation of the left-out participants using kendall’s tau, a non-parametric estimate of correlation that is less sensitive to outliers than a parametric correlation. This multivariate approach revealed normally distributed tau values with above chance prediction accuracy (i.e. Tau>0, t_(29)_=2.365, p=0.012, BF_+0_=4.13), albeit of modest effect size (d=0.432), supporting the notion that SI does indeed contain information that relates to the magnitude of donations in the hand condition. No differences were found between the self-reported mirror synesthete and the controls (t_(28)_=0.568, p=0.575, BF_10_=0.399). Result in **Fig. S4.**


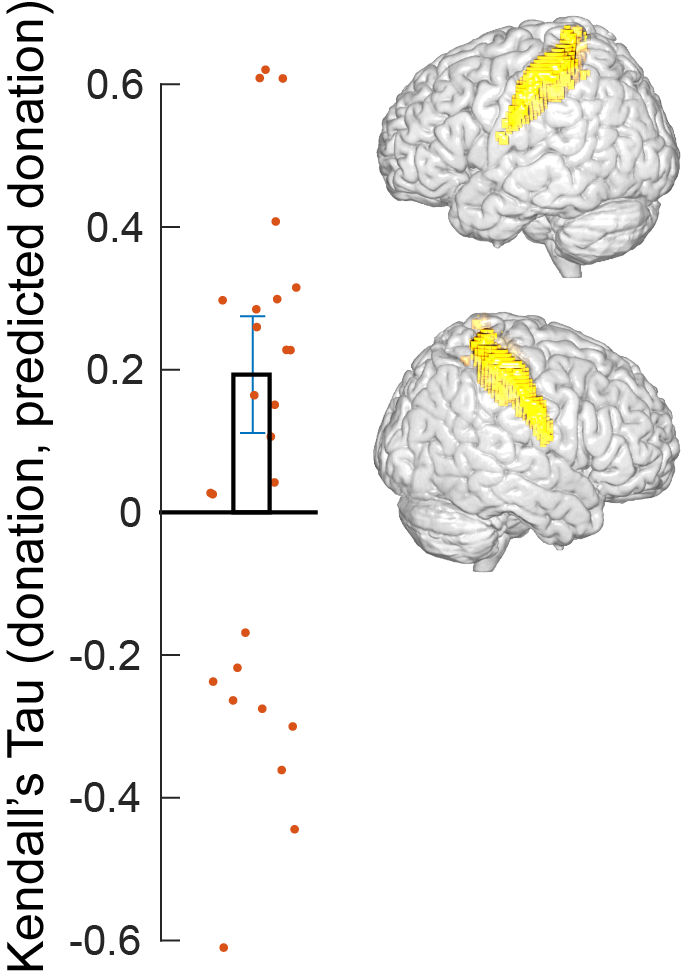


**Figure S4.** Mean (±sem) Kendall’s tau correlation between the actual donations of the participants and the ones predicted by a leave-one-out weighted partial least square regression based on parameter estimates from all SI voxels. Red dots indicate individual subject correlation. The two renders illustrate the location of the voxels included in the analysis based on the anatomy toolbox probabilistic maps of SI including BA 3a, 3b, 1 and 2.
